# Does bibliometric research confer legitimacy to research assessment practice? A sociological study of reputational control, 1972-2016

**DOI:** 10.1371/journal.pone.0199031

**Published:** 2018-06-14

**Authors:** Arlette Jappe, David Pithan, Thomas Heinze

**Affiliations:** 1 Interdisciplinary Center of Science and Technology Studies (IZWT), University of Wuppertal, Wuppertal, Germany; 2 Institute of Sociology, University of Wuppertal, Wuppertal, Germany; Institut Català de Paleoecologia Humana i Evolució Social (IPHES), SPAIN

## Abstract

The use of bibliometric measures in the evaluation of research has increased considerably based on expertise from the growing research field of evaluative citation analysis (ECA). However, mounting criticism of such metrics suggests that the professionalization of bibliometric expertise remains contested. This paper investigates why impact metrics, such as the journal impact factor and the h-index, proliferate even though their legitimacy as a means of professional research assessment is questioned. Our analysis is informed by two relevant sociological theories: Andrew Abbott’s theory of professions and Richard Whitley’s theory of scientific work. These complementary concepts are connected in order to demonstrate that ECA has failed so far to provide scientific authority for professional research assessment. This argument is based on an empirical investigation of the extent of reputational control in the relevant research area. Using three measures of reputational control that are computed from longitudinal inter-organizational networks in ECA (1972–2016), we show that peripheral and isolated actors contribute the same number of novel bibliometric indicators as central actors. In addition, the share of newcomers to the academic sector has remained high. These findings demonstrate that recent methodological debates in ECA have not been accompanied by the formation of an intellectual field in the sociological sense of a reputational organization. Therefore, we conclude that a growing gap exists between an academic sector with little capacity for collective action and increasing demand for routine performance assessment by research organizations and funding agencies. This gap has been filled by database providers. By selecting and distributing research metrics, these commercial providers have gained a powerful role in defining de-facto standards of research excellence without being challenged by expert authority.

## 1. Introduction

In recent years, the use of citation impact metrics has increased considerably. Some of these metrics are new, whereas some are variants or refinements of existing methods to measure the scientific impact of published research [1, 2]. A common aspect of these metrics is that they are based on citation data extracted from large multidisciplinary citation databases, most importantly the Web of Science (WoS) and Scopus. However, very few of the new impact metrics have actually been applied in research assessment practice; instead, bibliometric research assessment has been confronted repeatedly with criticism from academic communities, which perceive these assessments as threats to academic quality control via peer review [3–5]. Nevertheless, the simplest and most common metrics, the journal impact factor (JIF) [6] and the Hirsch Index (h-index or HI) [7] have spread widely among research administrators and funding agencies over the last decade [8, 9].

Citation impact metrics are used mainly by research organizations and research funding bodies in order to assess the performance of their programs, departments, groups, or individual researchers, as well as to monitor the activities of potential collaborators and competitors. The comparative literature on the governance of higher education shows that citation-based metrics have rarely gained a prominent place on the macro-level of national research funding systems [9–11]. Rather, their use as performance indicators can be understood as part of a broader set of organizational controlling techniques in response to changed expectations from their environment [12, 13]. In particular, the literature stresses increasing demands for accountability in national governance of public research and higher education, including international rankings and league tables that promote political discourse regarding global competition among research organizations for scientific prestige and talent [14–16]. More generally, these demands for accountability are viewed as a broader trend towards an audit society [17, 18], or as a manifestation of neoliberal ideology in the governance of higher education [19–21].

Concerning the potential negative effects of widespread application of citation-based performance metrics, scientists from different fields have voiced strong criticisms. On methodological grounds, citation indicators have been questioned by the International Mathematical Union [4]. Biologists and medical scientists claim that citation-based assessments could systematically underestimate the scientific value of certain research areas or activities, lending support to suboptimal resource allocations [3, 5, 22]. Similarly, citation-based funding could favor mainstream research and bias decisions against more original projects and interdisciplinary research [9]. On the individual level, performance indicators could induce the substitution of extrinsic motivations for intrinsic motivations, a problem referred to as goal displacement [9, 23]. In addition, citation metrics have been argued to be more likely to consecrate stable institutional prestige hierarchies than to foster inter-organizational competition [15, 24]. More generally, rankings in higher education are viewed as reactive, in that they create the social order they are supposed to measure [25]. These criticisms illustrate the contested nature of citation impact metrics among scientific stakeholders, but it is unclear as to what extent this contestation has impeded their diffusion in organizational decision-making.

**Fig 1:**
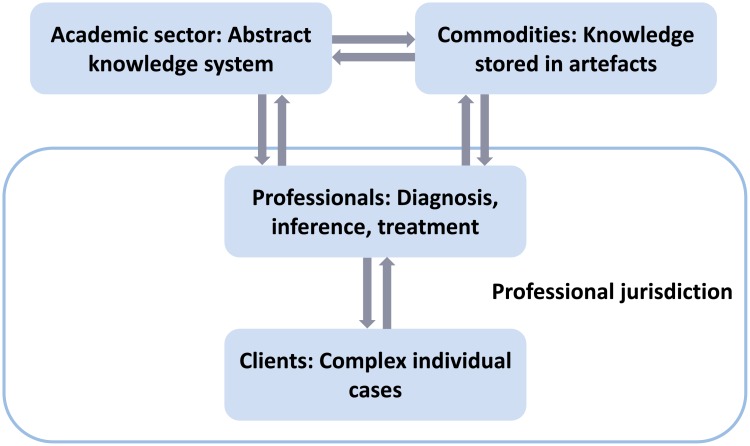
Theoretical framework of a mature profession according to Andrew Abbott. Visualization of the relationship between abstract knowledge and professional practice according to Abbott’s theory of professions [26, 27].

This paper is part of a larger project seeking to explain the proliferation of science metrics from the perspective of Andrew Abbott’s sociological theory of professions [26, 27]. This framework was chosen in order to investigate how particular methodological choices become socially established as professionally legitimate means of treating certain types of evaluation problems. Central to Abbott’s theory is a distinction between the professional work and the academic sector of a profession (Fig 1). This distinction describes a basic division of labor. As the academic sector develops abstract knowledge, the work of professionals and expert organizations consists of applying and using this abstract knowledge for the diagnosis and treatment of individual cases. Abstract knowledge is a source of legitimacy for professional work because it ties professional work to the general values of logical consistency, rationality, effectiveness, and progress. This scientific legitimacy includes a definition of the nature of problems, rational means of diagnosing them, and the delivery of effective treatment. In addition, abstract knowledge enables the instruction and training of students entering the profession and is oriented towards generating new mechanisms of diagnosis, inference, and treatment [26]. Thus, from the perspective of Abbott’s theory, scientific authority can be an important source of legitimacy in the competition among professional groups for exclusive domains of competence, so-called “professional jurisdictions”.

**Fig 2:**
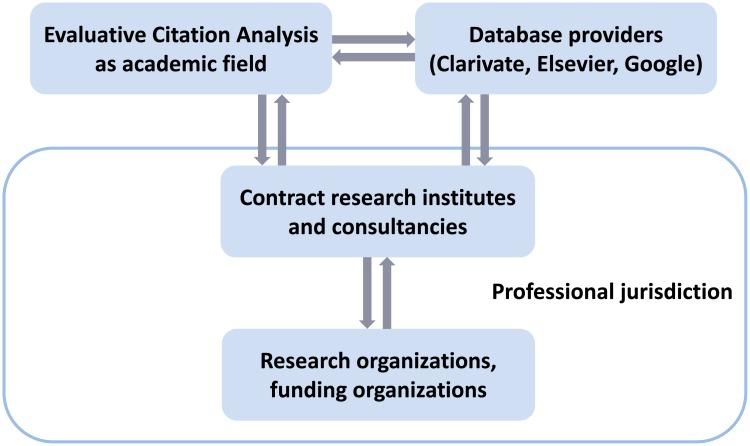
Bibliometric research assessment as an emerging profession. Visualization of the application of the theoretical framework in Fig 1 to the professional field of bibliometric research assessment.

Contrary to what would be expected from Abbott’s theory, we argue that, in the case of bibliometric assessment, the academic sector has failed to provide scientific authority for research assessment as a professional practice. This argument is based on an empirical investigation of the extent of reputational control [28] in the research area of evaluative citation analysis (ECA), an academic sector closely related to research assessment practice (Fig 2). As far as we are aware, the concept of reputational control has not been previously operationalized in the social science literature. This is an important gap because the concept is central to Whitley’s sociological theory of scientific work organization and the function of scientific disciplines [28, 29]. Our empirical investigation of reputational control involves several methodological steps. The starting point consists of the observation that a large number of novel citation impact metrics have been introduced in recent years as part of an intensified methodological debate in ECA. Based on bibliometric review, we collected 169 instances of new citation impact metrics since 1972, the year when Garfield introduced the JIF. In a second step, we identified all scientific literature in the WoS that cites any of these citation impact metrics and labeled this publication set ECA.

Reputational control was investigated with the help of longitudinal inter-organizational citation networks spanning the years 1972 to 2016. We identify organizations that are central or peripheral to these networks. Accordingly, we could trace whether proposals for new citation impact metrics were introduced from the network’s core or from its periphery. Furthermore, we determined whether new contributions introduced from the network’s core were more influential than those from its periphery and measured the extent to which newcomers entered the area over time. We found that a high percentage of contributions to ECA come from the citation network’s periphery and outsiders, though there are as many influential citation metrics from the network’s periphery as from the core. In addition, we identified a declining, yet still high, share of newcomers. Thus, our evidence indicates a low level of reputational control in ECA.

Based on our findings, we argue that the research area of ECA is not a coherent scientific field in the sociological sense of a reputational work organization [28]. ECA has always been open to contributions from outsiders and peripheral actors, suggesting that its social organization resembles a loose collection of scientific debates. The most significant example of an outsider contribution is the HI [7], which rapidly surpassed any previous metrics in terms of citation impact. The generally high share of outsider contributions indicates that ECA can be mastered with rather generalist scientific competencies, such as knowledge of statistical methods. In other words, contributing in a competent way to methodological debates on citation metrics and research assessments does not necessarily presuppose deep specialist skills that would be uniquely characteristic of ECA. Consistent with Whitley’s definition of the concept, openness to outsider contributions is a direct manifestation of weak reputational control.

As a corollary, though there are plenty of proposals on how to improve measurements of research impact, there is no coherent community of specialists to back up any of these claims with scientific authority. This article argues that, as a consequence of its open social structure and lack of reputational control, ECA confers little scientific authority to professional groups claiming exclusive competence in research assessment practice. Although attempts have been made to provide guidance on the appropriate selection of indicators [8, 30–32], no institutional mechanism exists for the effective communication and diffusion of scientifically accepted methodological recommendations to practitioners. As a consequence, relevant methodological issues, such as the scale-dependence of citation-impact [33], criticisms of the WoS subject categories as a taxonomy for field normalization [34–36], and the inadequacy of central tendency measures due to highly skewed distributions [37, 38], have not been effectively translated into assessment practice.

The present article aims to provide an explanation of why the academic research area of ECA has failed to provide scientific authority for quantitative research assessment. This explanation is “internalist”, in that it refers primarily to the cognitive and social structure of this particular research area. The present article draws a connection between Abbott´s theory of professional work and Whitley´s theory of scientific work. Yet, within Abbott´s framework, the academic sector is only one part of the picture (Figs 1 and 2). There are conceivable alternatives for how professional claims to expertise could gain acceptance in society. In evaluative bibliometrics, the role of professionals is usually performed either by individual researchers from the academic sector or by expert organizations, including contract research institutes or consulting firms. It is conceivable that these expert organizations take over the role to establish at least de-facto standards for bibliometric assessments. The work of professionals could be examined by identifying the individual experts and expert organizations that offer bibliometric assessment services and by investigating their activities and products over time. For example, Petersohn and Heinze [39] examined the history of the Centre for Science and Technology Studies (CWTS) at the University of Leiden (Netherlands), the leading expert organization in Europe. In addition, Petersohn [40] investigated jurisdictional claims by academic librarians in the United Kingdom and Germany. These established professionals increasingly provide bibliometric services to individual researchers and university management. Both studies are complementary to the internalist account of the present paper, in that they present rich detail on the institutional contexts in which new expert organizations (e.g., CWTS) could emerge and established professionals (e.g., librarians) could place social claims on the new jurisdiction of quantitative research assessment.

In line with Abbott, our findings should be combined with historical case studies of jurisdictional disputes over the development of research evaluation, particularly national contexts. However, it is not the aim of the present article to clarify the role of national governments in driving the demand for research metrics, though we recognize that this is an important issue. Thus far, a detailed historical account of the interaction between higher education policies and the development of bibliometric expertise has been provided only for the Netherlands [39]. The history of the UK Research Excellence Framework (REF) is another interesting case in point. Current evidence suggests that the introduction of research metrics was debated in 2006 in the UK but met with “fierce resistance” from within academia [41](p. 61). In the 2014 REF cycle, only 11 of 36 assessment units used citation data at all, but the relevant REF method document stresses that “no sub-panel will make use of JIFs, rankings or lists, or the perceived standing of the publisher, in assessing the quality of research outputs” [42](p. 8). Accordingly, in 2015, an independent group of experts concluded with reference to Cagan [3] that “across the research community, the description, production, and consumption of ‘metrics’ remains contested and open to misunderstandings” [43](p. viii). Apparently, the perceived lack of scientific legitimacy of ECA contributed to the British government’s decision to continue a national evaluation regime based primarily on peer review, despite criticisms of the heavy administrative burden that this regime imposes on the British public research and higher education system [44].

The structure of the article is as follows. First, we introduce Abbott’s [26, 27] theory of professions and Whitley’s [28, 29] theory of academic fields as reputational work organizations (Section 2). Second, we introduce data and methods, then present our variables and the delineation and partitioning of longitudinal inter-organizational citation networks into core-periphery structures (Section 3). Third, we present empirical results, starting with a basic characterization of contributions to the area of ECA, then examine the area’s growth and results pertaining to whether contributions to the area originated predominantly from the network’s core or from its periphery (Section 4). Finally, we summarize and discuss our findings (Section 5).

## 2. Theoretical considerations

In his theory of professions, Abbott [26, 27] argued that, in modern societies, professional expertise is provided in societally relevant problem areas in which either individual professionals or expert organizations apply abstract knowledge to complex individual cases. In fully established professions, such as medicine and law, the application of abstract knowledge includes diagnosis, inference, and treatment, and this three-fold professional practice is carried out in particular workplaces, such as hospitals or law firms. In their work, professionals and expert organizations often make use of specialized artefacts, including classifications, databases, expert systems, and other kinds of instruments or tools (Figs 1 and 2).

According to Abbott, it would be misleading to think of professionalization as a uniform development process. He argued that earlier process models in the sociology of professions failed to account for the historical variety in the evolution of professions. In search of a more open framework that could support a comparative research perspective, Abbott argued that the professions should be viewed as an interdependent system. From a system perspective, competition among professional groups takes center stage. Professional groups compete for recognition of their expertise and seek to establish exclusive domains of competence, which Abbott refers to as “professional jurisdictions”. In view of the historical variety in jurisdictional settlements in modern societies, the theory does not make specific assumptions of the role of the nation state, but argues for historical case studies of jurisdictions and jurisdictional disputes [26].

An important insight from Abbott’s theory is that professional work draws on two sources of legitimacy. First, because providers of different types of expertise often compete in the same societal problem area, they have to convince relevant actors in the public and legal arenas (government, parliament, courts, general public) to confer them legitimate social rights to conduct their professional work [26]. This requirement sometimes leads to a division of labor in which two professions have equal shares in a societal problem area, such as architects and engineers with respect to building houses. Another possibility is the subordination of a lower status profession to a dominant profession, such as nursing and medicine. Yet another and weaker form of legitimacy is advising or interpreting the actions of a dominant profession, such as when the clergy interprets the ultimate meanings of medically defined illnesses.

Second, the provision of professional expertise is firmly based on scientific legitimacy and, thus, depends on abstract knowledge, which is guided by the principles of logical consistency, rationality, and progress. This scientific legitimacy includes defining the nature of problems, rational ways of diagnosing them, and the delivery of effective treatment for clients. In addition, abstract knowledge enables the instruction and training of students entering the profession and is oriented towards generating new mechanisms of diagnosis, inference, and treatment [26].

Abbott emphasized that access to a professional jurisdiction requires the demonstration of scientific legitimacy. This requirement applies not only to the situation in which new experts submit claims for an entirely new jurisdiction, but also when they request entry to an existing one. The latter happened when psychiatry challenged criminality’s scientific authority and claimed that “the whole category system by which crime is classified, the typology of personal responsibility for action, was in fact a system about which psychiatrists knew more than lawyers” [26](p. 55). Therefore, the accreditation of legitimate social rights to a new type of professional work depends on the challenger’s ability to demonstrate that a new type of abstract knowledge has higher scientific legitimacy than that of an existing profession.

The question remains as to when one academic field has higher scientific legitimacy than another field, and under which circumstances an academic field would have such legitimacy. These questions can be answered considering Whitley’s [28, 29] theory of reputational work control. He argued that academic fields differ in their capability to (a) establish standards of research competence, work procedures, and methodical skills; (b) develop highly structured and formalized languages that enable researchers to communicate their results and co-ordinate their efforts; and (c) provide socially prestigious scientific reputations [28]. According to Whitley, academic fields exert strong reputational control over their members “when they control the acquisition of standardized skills which are necessary to produce acceptable task outcomes, and when they control a standardized symbol system which monopolizes the communication of results and the means of obtaining reputations” [28](p. 32). In contrast, academic fields exert limited reputational control over their members when they have to “share the evaluation of task outcomes with other scientific or non-scientific groups, where techniques are not highly standardized and exclusively controlled, and reputations are not highly valued” [28](p. 43).

Whitley’s differentiation between strong and limited reputational control manifests itself in the social closure of academic fields to contributions by outsiders. Although “each field has to have some particular skills, which excludes outsiders and enables results to be compared and evaluated in terms of their significance for collective goals” [28](p. 31), fields with strong reputational control exclude outsiders more effectively than fields with limited reputational control that have more permeable and loose boundaries. These latter are sometimes even invaded by fields with strong reputational control, such as physics in the natural sciences and economics in the social sciences [28]. Social closure of fields by their scientific members may be unintended and may, at times, have disadvantages for the progress of knowledge, but it is a predictable and observable effect of strong reputational control.

Thus, following Whitley, the assumption that academic fields furnish scientific legitimacy to different degrees involves both cognitive and social aspects. On the cognitive side, pursuing collective goals that are achievable only with highly field-specific competencies, including highly formalized communication standards, implies the achievement of a certain level of consensus on acceptable methodologies. Reference to such consensus among a community of specialists is a suitable way to convey scientific legitimacy to professional work. On the social side, scientific fields with strong reputational control also tend to be fields that have higher collective reputations or higher status in the eyes of other academic fields and, more generally, of society. Following Whitley, reference to expertise in high-status fields, such as physics or economics, is more likely to convey scientific legitimacy in societal arenas than reference to fields with lower status.

Our argument does not aim to extend or contest Abbott’s or Whitley’s theories, but combines the two theories where they best fit to develop a conceptual framework capable of guiding empirical analysis. This effort aims to improve our sociological understanding of how internal field structures affect the opportunities for bibliometric professionals and expert organizations to achieving accreditation in legal and public arenas. Extending and contesting theories is not a goal in itself. Rather, fruitfully combining existing theoretical contributions and applying them to new empirical phenomena (e.g., the new profession of bibliometric experts) is as important and interesting for a broad sociological readership as the revision, or even replacement, of existing theories.

Thus, Abbott’s and Whitley’s ideas are used to derive three propositions. First, the capability to legitimize expertise as professional work in competitive public and legal arenas depends on scientific legitimacy. Second, from a sociological perspective, scientific legitimacy is a function of reputational control, as strong reputational control confers scientific legitimacy to a greater extent than limited reputational control. Third, the closure of academic fields to outsider contributions is a useful indicator of reputational control.

## 3. Material and methods

### 3.1. Evaluative citation analysis as a research area

Different methodological approaches are used to identify research fields on the basis of bibliometric data, including delineations by search terms or publications in key journals. The content-based approach of the present study differs from the methods for field delineation often found in the scientometric literature, as they frequently rest on a fixed list of journals or eminent authors for a given period. This section provides contextual information on ECA and the most widely used citation impact metrics.

The research specialty of evaluative bibliometrics has been studied repeatedly with scientometric mapping techniques [45–49]. This literature locates the area within the broader disciplinary category of library and information sciences [50–54]. Different methodological approaches have been pursued to identify substructures based on delineations, such as search terms or publications in key journals. These studies converge in ECA being a core bibliometric research area since the introduction of the Science Citation Index (SCI) in the 1960s; as such, it can be distinguished from “visualization of knowledge domains” and “webometrics” [45, 47]. In addition, “impact factor,” “h-index,” and “journal impact” are core keywords associated with citation analysis [50].

The history of ECA as a research area is closely tied to the creation of the SCI as the first multidisciplinary citation database [55, 56]. The SCI started with approximately 600 journals in 1964, and by 1972 the number had grown to over 2400 [6]. Together with other related citation databases, including the Social Science Citation Index (SSCI) and the Humanities and Arts Index (AHI), the SCI has been the main tool for ECA.

The two most influential citation impact indicators are the JIF, published by Eugene Garfield in 1972 [6], and the HI, published by Jorge E. Hirsch in 2005 [7]. Precursors of the JIF include Garfield’s [57] suggestion that counting references to journals could measure the impact of a journal, and the expression “impact factor” was used for the first time by Garfield and Sher [58], who described the project to construct the SCI and the data that it should provide. The JIF of a journal is calculated as the “sum of the citations received in a given year by the articles published in the journal in the previous 2 years divided by the number of articles published in the journal in the previous 2 years” (for a formal notation, see [1](p. 228)).

The originality of the JIF does not consist of the mathematical formula as such, but rather in the broad claim to measure journal rankings covering “the whole of science and technology” [6](p. 472). Based on a systematic analysis of citation patterns of more than 2000 journals, then covered by the SCI, Garfield expressed the vision that, “perhaps the most important application of citation analysis is in studies of science policy and research evaluation,” and that, “using the SCI database to map the journals citation network will contribute to more efficient science” [6](p. 476). Since 1975, the JIF has been distributed via the annual publication of Journal Citation Reports. The SCI, together with the SSCI and AHI, held a monopoly on publication and citation data until 2004, when the publishing house Elsevier and Google introduced their own databases (Scopus and Google Scholar, respectively).

A researcher’s HI is defined as “*h*, if *h* of his/her *P* articles received at least *h* citations each and the rest (*P-h*) articles have received no more than *h* citations each” (for a formal notation see [1](p. 162)). The HI was the first citation indicator to surpass the JIF in terms of both total citation frequency and the number of follow-up inventions (see below). At first look, the HI itself can be regarded as a follow-up invention inspired by JIF. Yet, the HI is also an original contribution that quickly unleashed a new stream of research that has expanded so quickly that it is now clearly distinguishable from the earlier research area on citation impact. The most recent Handbook of Bibliometric Indicators explicitly distinguishes bibliometric indices in “the era before and the era after the Hirsch Index” [1](p. 162).

Interestingly, Jorge Hirsch has always been an outsider to ECA. Thus far, he has declined to present his influential invention at one of the biannual Science and Technology Indicator Conferences. However, his behavior should not be interpreted as an indication that ECA does not exist as a research area or that bibliometricians do not seek professional accreditation. Rather, Hirsch’s behavior showcases the permeable boundaries characteristic of ECA, a finding that will be examined empirically in more detail below.

### 3.2. Citation impact metrics as “follow-up inventions”

Following Whitley’s [28, 29] theory of scientific work, we are particularly interested in how reputational control over the publication and dissemination of novel intellectual contributions is exerted in ECA. Therefore, instead of taking all possible publications of ECA into account, we distinguished between (a) publications that introduced novel bibliometric indicators that have received an extraordinarily high number of citations and, thus, have defined the research area of ECA (“breakthroughs”), (b) publications that elaborate on or improve these breakthroughs and, thus, have followed in the footsteps of the breakthroughs without becoming highly influential themselves (“follow-up inventions”), and (c) publications that cited either (a) or (b) or both (“follow-up research”).

The methodological distinction between research breakthroughs and follow-up research was introduced by Heinze et al. [59], who studied growth patterns of publications that cited two Nobel Prize–winning contributions in chemistry and physics. They focused on all publications that cite the papers in which these two particular research breakthroughs were first published. However, their method excluded follow-up inventions that elaborate on or improve these breakthroughs but do not cite the particular papers in which they were first published. Therefore, we developed a more comprehensive approach that takes these follow-up inventions into account. Typically, they can be identified by reference to review papers and handbooks. Therefore, our focus is on all follow-up publications that cite both breakthroughs and follow-up inventions.

Methodologically, we identified the JIF and HI as the “breakthrough” indicators in the research area of ECA. Both JIF and HI have inspired the publication of a large number of bibliometric indicators that elaborate on or improve them and have followed in their footsteps without becoming highly influential themselves. Thus, an important step in our research consisted of the compilation of a comprehensive list of these follow-up inventions based on the review literature. All follow-up inventions from 1972–2004 were categorized as related to JIF, whereas follow-up inventions after 2004 were classified as either JIF-type or HI-type in broad domains within ECA.

The following literature was used to identify JIF-related follow-up inventions: handbooks [1, 60], review articles [2, 61–66], and literature sections of research articles [67–75]. The set of JIF-related follow-up inventions comprised a total of 74 publications proposing new indicators (S1 Table). The following literature was used to identify HI-related follow-up inventions: the handbook [65], literature reviews [62, 76–82], and literature sections of research articles [83–90]. The set of HI-related follow-up inventions comprised a total of 95 publications proposing new indicators (S2 Table).

The following criteria were used in the selection of relevant citation impact metrics. First, our selection includes new bibliometric indices that were designed for research evaluation purposes and published in the WoS, the database platform that includes the SCI. We included citation metrics for journals as publishing outlets, as well as metrics for productive units of the science system, such as individual scientists, research groups, research organizations, funding programs, and countries. These are the units that are most relevant for evaluation purposes. Second, complementary metrics that are not directly related to JIF and HI were excluded, such as publication output counts, metrics of collaboration, internationalization, bibliographic coupling, citation entropy, interdisciplinarity, gender segregation, input-related metrics, patent citations, or altmetrics [1]. Third, publications that criticize, review, or apply existing citation impact indices without presenting new indicators themselves were excluded.

There was not always a clear boundary between a “new bibliometric indicator” and yet another “variation of the same.” This problem is inherent to our approach because follow-up inventions are not required to be highly original by definition. Therefore, we applied a pragmatic approach by including all publications from the aforementioned review literature that claimed to introduce a new bibliometric indicator in their title, abstract, or main text. In some cases, more than one publication claimed to introduce the same citation index (so-called “multiples” [91]), either because the same authors published it in different journals or because different authors published the same idea. In both cases, the earlier publication was included in our set of follow-up inventions.

After identifying two breakthroughs and 169 follow-up inventions, we retrieved all publications that cite these 171 publications between 1972 and 2016. This set of follow-up research defined the evolving research area of ECA for the subsequent network analysis. Publications were downloaded from the core collection of the WoS, including articles, notes, reviews, letters, and proceeding papers. The set of JIF-related follow-up research included 2821 publications (1972–2016), and HI-related follow-up research included 2437 publications (2005–2016).

### 3.3. Core and periphery in the organizational field of ECA

We chose to study inter-organizational networks instead of author networks in order to separate network centrality from academic seniority. Recent studies have shown that, among all authors covered by citation databases, more than half publish only one item. Ioannidis, Boyack, and Klavans [92] found that 58% of unique author identifiers in Scopus (1996–2011) have papers published only in a single year, whereas Moody [93] found that 66% of authors of English-language articles listed in Sociological Abstracts (1963–1999) appear only once. It can be assumed that a significant share of these one-time authors are students who publish results from MA/MSc or PhD work, but more systematic information on the academic age of authors is not available. We are interested in professionalization, so we grouped these young academic authors with their faculty so that network centrality would not presuppose academic seniority. Selecting the upper organizational level for the study of citation networks is also in line with Abbott’s [26, 27] theory, which argues that organizations as employers of scientists produce and cultivate academic knowledge, including the training of students, that is used and applied by individual professionals and expert organizations. Our unit of analysis is the upper organizational level, typically universities, but also non-university research institutions, and in some cases companies. Relevant examples are: University of Copenhagen, University of Toronto, Louisiana State University, University of Chicago, University of Indiana, National Bureau of Economic Research, Max Planck Institute for Solid State Research, Computer Horizons, Elsevier, and Thomson Reuters, formerly ISI Institute Philadelphia, today Clarivate Analytics.

Studying social networks from a longitudinal perspective has become increasingly common [94], and many analyses focus on the structural characteristics of such networks and their change over time, particularly with algorithm-generated artificial networks whose mathematical properties are then further examined with sophisticated statistical models [95, 96]. However, to the best of our knowledge, longitudinal inter-organizational citation networks have not been examined thus far. Therefore, when we delineated our networks, we consulted studies with a somewhat different focus, including the use of multi-modal networks to model scientific fields [97] and the study of inter-institutional collaboration patterns in the life sciences and biotechnology [98].

First, the data had to be cleaned. For this purpose, a thesaurus was constructed based on manual cleaning of organizational homonyms in the address field of publications. The nodes are unique organizational entities. Our entire dataset contains N = 2.499 organizations. The edges are citations from these entities to other entities within the publication set. We used whole counts, as opposed to fractional counts, and each organization in the address field was counted with a weight of 1 (see below). Second, the longitudinal network analysis was based on overlapping 5-year slices to ensure that fluctuations in citations from year to year were smoothed. For example, the 1972–76 network captures all organizations and the respective citations among each other in the years 1972 to 1976. The next slice is the 1973–77 network, which contains all organizations and the respective citations among each other in the years 1973 to 1977. This pattern continues up until 2012–16. Five-year citation windows are favored over smaller windows (e.g., 2-year or 3-year) in the literature because they better capture citation distributions and are robust regarding full versus fractional counting techniques [99, 100]. Third, we used software tools to construct all citation networks. The WoS raw data were parsed and analyzed using BiblioTools [101], and we wrote Python scripts to construct network matrices [102].

As a next step, we delineated a core-periphery structure for each 5-year citation network. Structures were delineated separately for the JIF-related networks (1972–2016) and HI-related networks (2005–2016). The concept of core-periphery structures is widespread in social network analysis, but no standard formal conceptualization for partitioning networks into core and periphery has emerged thus far [103, 104]. Multiple ways of assessing such structures have been proposed, including centrality-based concentration measures [105] and cohesive subgroups [106].

All existing approaches presuppose a core of nodes that not only have a high indegree (i.e., a high number of citations received), but are also strongly connected among each other. However, citation distributions are typically highly skewed, with few papers receiving many, if not most, citations and a long tail of publications receiving only a few mentions [37, 38, 103]. Therefore, mutual connections between nodes with a high indegree may not occur as often as in other types of social networks, such as interlocking directorates [107, 108]. Consequently, a measure for ordering and comparing the skewed indegree distribution was needed. We applied the method of characteristic scores and scales (CSS), an approach developed to define groups of ranked observations in highly skewed distributions [109, 110].

CSS divides the indegree distribution of citation networks into classes without any preset thresholds, using cut-off parameters, such as the arithmetic mean, instead. In a first step, the application of the arithmetic mean to the indegree distribution yields two groups: below mean (CSS = 1) and above mean. In a second step, the above mean group is further divided by the arithmetic mean within that group, yielding another two groups: below mean (CSS = 2) and above mean (CSS = 3). In this way, CSS generates three groups that can be interpreted in terms of core and periphery. CSS = 1 can be regarded as the periphery, whereas CSS = 3 can be regarded as the network core. CSS = 2 stands between the periphery and the core and is interpreted as semi-periphery because organizations of this group have indegrees in between the first and second iterations.

The recent literature suggests that, in very large citation networks, fractional counting could lead to different results at the author level than whole counts [111]. Therefore, we compared the CSS distribution of our inter-organizational citation network for both whole and fractional counts. We found that both counting methods yield very similar results (available upon request from the corresponding author). Our findings corroborate [100] that 5-year citation windows are robust regarding fractional and whole counting. In addition, the aggregation of citations at the organizational level further alleviates differences between the two counting methods.

### 3.4. Variables for reputational control in ECA

Ordering the indegree distribution of citation networks via CSS allowed us to operationalize Whitley’s concept of reputational control. As mentioned above, distinct scientific competencies, work procedures, and methodical skills separate academic fields with strong reputational control from one another. Thus, to the extent that field-specific scientific competencies, work procedures, and methodical skills are required to contribute to ECA, they constitute an entry threshold for outsiders; here, they are organizations from the field’s periphery.

Three variables were defined to investigate entry thresholds to the field. First, by transforming the indegree distribution of longitudinal inter-organizational citation networks in CSS groups, we examined the number of follow-up inventions (JIF: n = 74; HI: n = 95) that originated from either central or peripheral positions in the network. Second, we examined the annual number of citations that follow-up inventions from the network’s core and periphery received in later periods. A high share of follow-up inventions from the periphery that received a high annual number of citations in later years would indicate low entry thresholds and, therefore, limited reputational control. In contrast, a high share of follow-up inventions from the core that received a high number of annual citations in later years would indicate effective entry thresholds and, therefore, strong reputational control. Third, we counted the share of newcomers and multiple contributors to the field. Newcomers were defined as organizations that contribute to the stream of follow-up research for the first time in the respective year. Multiple contributors were defined as organizations that have already published follow-up research in at least 5 years, including the given year. A large share of newcomers would indicate low thresholds for field entry and, thus, limited reputational control. In contrast, a large share of multiple contributors would indicate higher thresholds for field entry and, thus, stronger reputational control.

## 4. Empirical results

**Fig 3:**
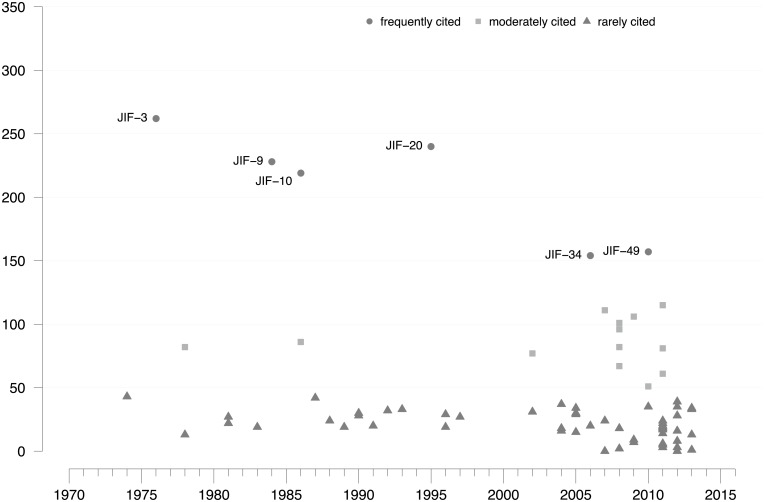
Citation distribution of JIF-related follow-up inventions. Citation frequencies for 74 JIF-related follow-up inventions. Citation partition (frequently, moderately, rarely) derived using CSS. Frequently cited indicators are labeled with IDs from S1 Table. Citation window: year of publication –2016. Source: Web of Science.

Regarding JIF, 74 publications were identified as follow-up inventions, 16 (22%) of which cite Garfield’s work in 1972 [6]. The number of inventions, and thus indicator development, has sharply increased since the mid-2000s, following the publication by Hirsch [7]. Twenty-seven new indicators were published over 33 years (1972–2004), but 47 new indicators were published over the subsequent 10 years (2005–2014). Putting this growth into perspective, if JIF-related indicator development continued to grow after 2005 at the same speed as before, only eight new indicators would have been published. As a measure of visibility and scientific impact, Fig 3 shows the citation frequency of each JIF-related follow-up invention since the year of its introduction. The most frequently cited publications, based on a CSS partition of the total number of citations, are (in descending order): JIF-3, JIF-20, JIF-9, JIF-10, JIF-49, and JIF-34.

Three of the six frequently cited indicators belong to the discussion of *indirect citation metrics*. This area includes the influence weight of citing units (JIF-3); the impact-adjusted citations approach (JIF-9), which has been widely used to rank the relative influence of economic journals; and the weighted PageRank (JIF-34), which is based on Google’s PageRank algorithm. Another two frequently cited indictors are located in the topical area of *field normalization*, which deals with indicators based on average citation counts: the relative indicators approach (JIF-10) and the field normalized citation score (JIF-20). Another frequently cited indicator, the source normalized impact per paper (JIF-49), is part of the topical area of *source normalization* or *fractional citation weighting*.

**Fig 4:**
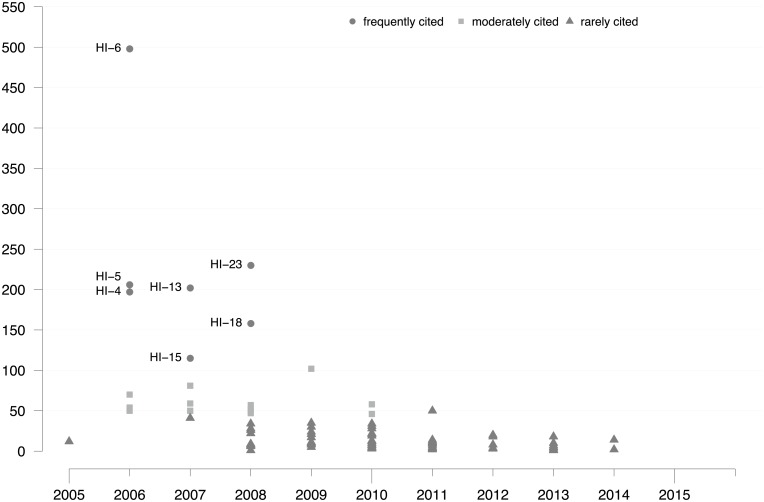
Citation distribution of HI-related follow-up inventions. Citation frequencies for 95 HI-related follow-up inventions. Citation partition (frequently, moderately, rarely) derived using CSS. Frequently cited indicators are labeled with IDs from S2 Table. Citation window: year of publication –2016. Source: Web of Science.

Regarding HI, 95 publications were identified as follow-up inventions, 90 (95%) of which cite Hirsch [7]. Compared to JIF, there were twice as many HI-related follow-up inventions in the same time period, indicating extremely high interest in the growing ECA community in developing improved HI-related citation metrics. As a measure of visibility and scientific impact, Fig 4 shows the citation frequency of each HI-related follow-up invention since the year of its introduction. The most frequently cited publications based on a CSS partition of the total number of citations were (in descending order): HI-6, HI-23, HI-5, HI-13, HI-4, HI-18, and HI-15.

Three of the seven frequently cited indicators have contributed to a discussion on *HI’s insensitivity to highly cited papers*, also called *citation intensity of the h-core*. The g-index (HI-6), R index (HI-13), and m-index (HI-18) are meant to improve the HI. Furthermore, *HI’s dependence on academic age* and *co-authorship issues* are addressed by HI-15 and HI-4. Another frequently cited indicator, the Hirsch-type index for journals (HI-5), extends the HI to a different aggregation level. Finally, there is one frequently cited contribution to the topical area of *field normalization*, the cf indicator (HI-23).

This brief introduction of the 13 most frequently cited follow-up inventions does not cover the entire thematic spectrum of JIF-related or HI-related follow-up research. More detailed information on thematic differentiation in the two publication sets of follow-up inventions is available in the review literature (S1 and S2 Tables).

The sharp increase in new bibliometric indicators since 2005 has led to a similarly sharp increase in the number of publications citing the two breakthroughs, their follow-up inventions, or both (Fig 3). Notably, two markedly different growth periods were discerned. From 1973 to 2005, there was moderate expansion of JIF-related follow-up research, whereas the number of citations of Garfield [6] remained fairly constant. Yet, after 2005, there was a steep increase in JIF-related follow-up research at the time when HI-related follow-up research set in. Since 2007, there have been more HI-related follow-up publications per year than JIF-related follow-up publications. These results suggest that the above-average dynamics in JIF-related follow-up research has been ignited primarily by the strong interest in the HI.

As mentioned above, Hirsch contributed his invention [7] as an outsider to the research field. Publications citing the HI account for 54% of all follow-up publications and 21% of JIF-related follow-up publications in the period 2012–2016. Furthermore, an intensifying methodological debate on topics, such as indirect citation indices, field normalization, percentile indices, and source normalization [2], has contributed to the growth of JIF-related follow-up research. The large number of new indirect citation metrics (e.g., JIF-34 or JIF-39), and metrics that are distributed online (e.g., HI, JIF-39, JIF-40, JIF-49, JIF-67), indicates that the soaring interest in ECA is supported by technological factors, such as increased computing capacity and expansion of the world wide web. Taken together, the data shown in Figs 1–3 suggest that, since the mid-2000s, a strong drive to apply online metrics, especially the HI, has unsettled the rather moderate growth in the three previous decades.

**Fig 5:**
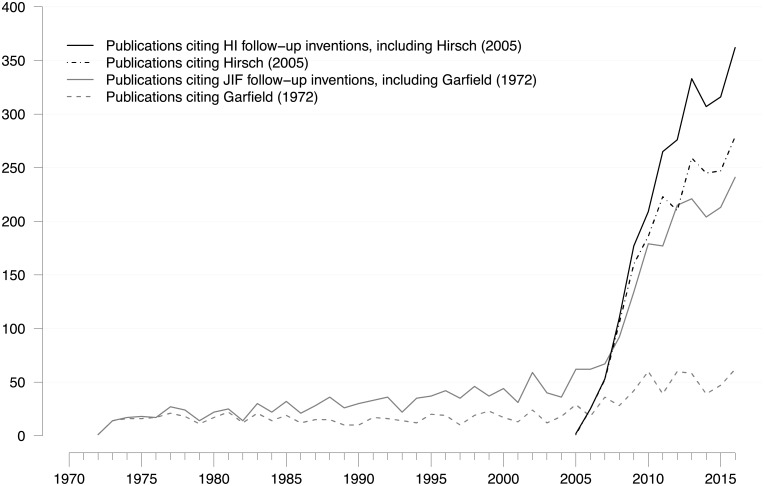
Growth of JIF-related and HI-related follow-up research. Annual number of articles, notes, reviews, letters, and proceeding papers citing a) HI-related follow-up inventions, b) the HI breakthrough, c) JIF-related follow-up inventions, and d) the JIF breakthrough. Citation window: 1972–2016. Publications that cite more than one breakthrough or follow-up invention were counted only once. Data source: Web of Science.

To substantiate these claims, we checked three alternative explanations: mutual overlap, database expansion, and the introduction of Scopus and Google Scholar. Regarding the first alternative, publications from the two publication sets sometimes cited follow-up inventions from the other set; yet, the number of these co-citations was not sufficiently large to explain the expansion of JIF-related follow-up research. In the period 2012–2016, an average 37% of JIF-related follow-up publications cited HI-related follow-up indicators, including the HI (n = 95). Second, the steep growth in JIF-related follow-up research also cannot be explained by general expansion of the publication database. As the SCI expanded substantially between 1970 and 2010, its growth rate remained relatively stable at 2.7% per year, which is equivalent to a doubling time of 26 years [112]. In contrast, the number of JIF-related publications increased by a factor of 7 in the period 1975–2010 and by a factor of 10 in the time period 1975–2015 (Fig 5). Third, we probed to what extent “Scopus” and “Google” were covered in the titles and abstracts of our publication sets. Regarding “follow-up inventions”, they were mentioned in 9 JIF-articles (n = 74) and 5 HI-articles (n = 95), and regarding “follow-up research”, we identified 110 JIF-articles (out of 2.821) and 190 HI-articles (out of 2.437), totaling 6% of all articles in the combined publication sets. If “Scopus” and “Google had been a major reason for the rapid growth after 2005, many more articles should have been found.

A second notable result is depicted in Fig 5. A publication set based on citations to Garfield [6] alone, and not including its follow-up inventions, severely underestimated the influence that this breakthrough had on the later development of ECA. The grey dashed line represents the number of citations to Garfield [6], whereas the grey solid line includes the number of citations to either Garfield [6] or related follow-up inventions (n = 74). In the period 1972–2004, the publication set including JIF-related follow-up inventions is approximately twice the size of the volume of citations to Garfield [6]. In the period 2005–2016, the gap between the two sets widens considerably, with the publication set including JIF-related follow-up inventions being up to four-times the size of the volume of citations to Garfield [6]. This is not to say that all follow-up inventions should ultimately be credited to Garfield as the pioneer in the field; rather, this gap indicates that many scientists felt inspired by the idea and saw a need for the original concept to be altered. Therefore, they continued to work on JIF-related topics and cited more recent contributions rather than the breakthrough. In other words, follow-up inventions are an important manifestation of a long-term research impact that would be missed if citations to the breakthrough alone had been examined.

Regarding the three variables that measure reputational work control in the research area of ECA, we first examined the number of follow-up inventions (JIF = 74, HI = 95) that originated from central or peripheral positions in citation networks. Table 1 shows the number and percentage of follow-up inventions from core, semi-peripheral, peripheral, and isolated organizations in the two longitudinal citation networks. The four partitions of the citation networks were determined using CSS [109], and they are based on the organization’s CSS partition in the 5-year period prior to the publication of follow-up inventions. Therefore, we measured organizational positions in citation networks before they contributed a particular follow-up invention.

**Table 1:** Network position and follow-up inventions in evaluative citation analysis.

Follow-up inventions published	JIF-related	HI-related	Total
1972–2004	27	--	--
2005–2014	47	95	142
By core organizations (CSS = 3)			
1972–2004	9 (33%)	--	--
2005–2014	16 (34%)	28 (29%)	44 (31%)
By semi-peripheral organizations (CSS = 2)			
1972–2004	2 (7%)	--	--
2005–2014	10 (21%)	22 (23%)	32 (23%)
By peripheral organizations (CSS = 1)			
1972–2004	6 (22%)	--	--
2005–2014	10 (21%)	18 (19%)	28 (20%)
By isolated organizations (CSS = 0)			
1972–2004	10 (37%)	--	--
2005–2014	11 (23%)	27 (28%)	38 (27%)

If follow-up inventions originated from more than one organization, the highest CSS value was used. Citation window: 1972–2016. Data source: Web of Science.

Overall, roughly one-third of all follow-up inventions originated from the core of the network. In contrast, between 45 and 50% of all follow-up inventions originated from the network’s periphery or isolated organizations. Though the share of isolated organizations was even higher for JIF-related inventions during the slow growth period (1972–2004), there was not much difference between the JIF-related and HI-related follow-up inventions during the fast growth period (2005–2016). These results are consistent with one of the two breakthroughs originating from an outsider [7] and suggest limited reputational control.

Second, we examined the visibility and scientific recognition of JIF-related and HI-related follow-up inventions that originated from central or peripheral positions in the longitudinal citation networks. Table 2 shows the number of follow-up inventions that received above- or below-average citation scores and were published by core, semi-peripheral, peripheral, or isolated organizations. Again, the four partitions of the citation networks were determined using CSS [109] and are based on the organization’s CSS partition up to 5 years prior to the publication of their follow-up inventions. Therefore, we operationalized reputational control in the sense that outsider contributions may be expected to receive less visibility and scientific recognition than contributions by renowned field experts.

**Table 2:** Network position and annual citation scores for JIF/HI-related follow-up inventions.

	Number of follow-up inventions in stratified citation frequency partition		
Organization’s position in citation network prior to publication of its follow-up invention	Frequently cited (CSS = 3) JIF/HI/JIF+HI	Moderately cited (CSS = 2) JIF/HI/JIF+HI	Rarely cited (CSS = 1) JIF/HI/JIF+HI
Core (CSS = 3)	3 / 3 / 6	5 / 3 / 8	17 / 22 / 39
Semi-periphery (CSS = 2)	0 / 1 / 1	2 / 2 / 4	10 / 19 / 29
Periphery (CSS = 1)	3 / 2 / 5	4 / 3 / 7	9 / 13 / 22
Isolates (CSS = 0)	0 / 1 / 1	2 / 8 / 10	19 / 18 / 37
Total	6 / 7 / 13	13 / 16 / 29	55 / 66 / 121

Citation windows: 1972–2016 (JIF), 2005–2016 (HI). Network position for organizations in HI-related citation networks for the years prior to 2010 were calculated using total (JIF+HI) network data. Data source: Web of Science.

Overall, approximately 46% (6 out of 13) of all frequently cited follow-up inventions originated from the core of the network (JIF: 50%; HI: 43%). Similarly, approximately 46% of all frequently cited follow-up inventions originated from the network’s periphery or isolated organizations (JIF: 50%; HI: 43%; Table 2). In contrast, approximately 32% of all rarely cited follow-up inventions originated from the core of the network (JIF: 31%; HI: 33%), whereas approximately 49% of all rarely cited follow-up inventions originated from the network’s periphery or isolated organizations (JIF: 51%; HI: 47%; Table 2). The remaining percentage of rarely cited follow-up inventions originated from the semi-periphery. Thus, the two domains are similar regarding the citation frequency of inventions originating from the core and periphery.

The emerging pattern is consistent with organizations in the network’s periphery and isolated organizations contributing the same number of influential (i.e., frequently cited) follow-up inventions as organizations in the network’s core. The correlation between the two variables is 0.14, indicating almost no relationship between where the new bibliometric indicators were developed (core or periphery) and how they were received in the field (frequently or rarely cited). Rather, over more than 40 years, influential follow-up inventions were regularly introduced by organizations with a peripheral or isolated position in the knowledge network. However, it would be wrong to conclude from these findings that ECA does not exist as a research area, as its extraordinary growth shows its vitality and attractiveness for many researchers. Therefore, we conclude that reputational control was, and still is, rather limited in this research field.

**Fig 6:**
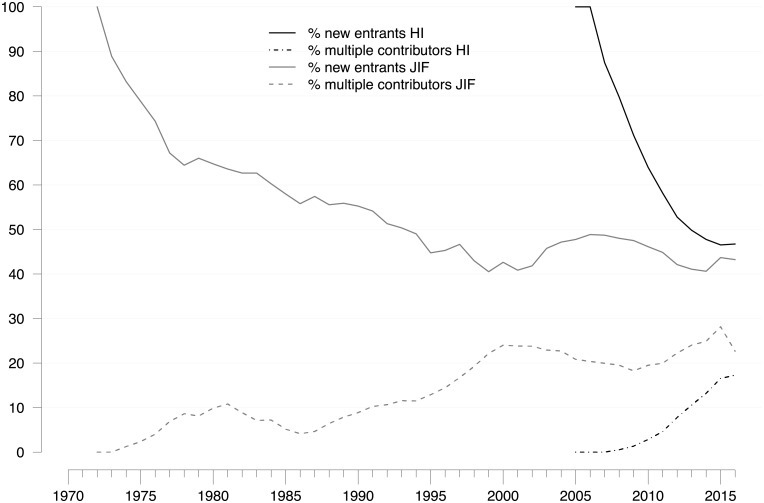
New entrants and multiple contributors to JIF-related and HI-related research. Percentage of (research) organizations that, in a given year, either entered the publication set for the first time (new entrants) or contributed publications in at least five different years (multiple contributors). Five-year moving averages are shown. Citation window: 1972–2016. Data source: Web of Science. A high annual share of new entrants indicates a low threshold for field entry and limited reputational control, whereas a high share of multiple contributors indicates a higher threshold for field entry and stronger reputational control.

We also considered the extent to which newcomers entered both research areas and, conversely, how many multiple contributors were operative. As Fig 6 illustrates, the share of newcomer organizations decreased over time, more rapidly so in HI-related research, but has remained at a relatively high level; between 40 and 50% of all follow-up publications originated from organizations contributing to the field for the first time. Similarly, though the share of multiple contributors continuously increased, suggesting some degree of consolidation, it does not come close to the level of newcomers. These findings suggest that the field has permeable boundaries. Therefore, we conclude that reputational control was, and still is, quite limited in both JIF-related and HI-related follow-up research.

Thus far, the most influential outsider contribution was the HI [7], which rapidly surpassed the original JIF in terms of citation frequency and follow-up inventions. To assess the HI’s effect on the existing JIF citation network, we determined the modularity index that measures the extent to which networks are fragmented into clearly separable cliques [113]. As high modularity scores indicate internal fragmentation, low values indicate a more compact and interconnected network.

**Fig 7:**
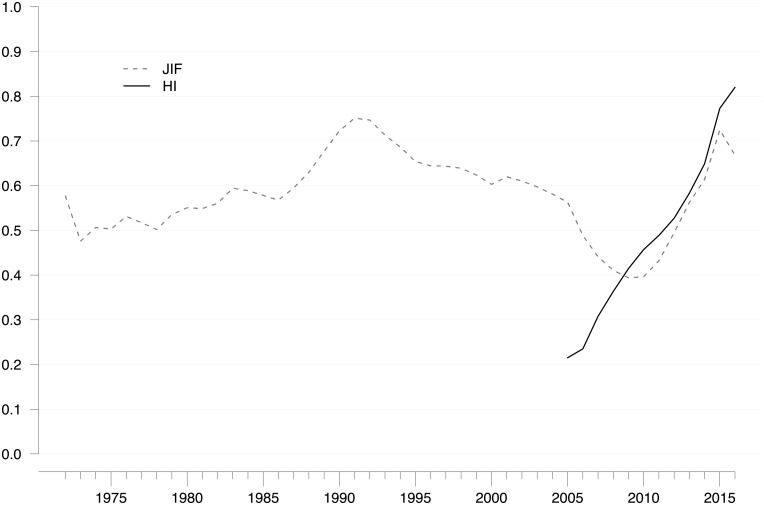
Fragmentation of JIF-related and HI-related research. Annual score for the modularity index in JIF/HI-related citation networks (based on follow-up research). Five-year moving averages are shown. Citation window: 1972–2016. Data source: Web of Science. High modularity scores indicate internal fragmentation, whereas low values indicate a more compact and interconnected network.

As Fig 7 shows, the JIF citation network became more compact and cohesive during the 1990s and 2000s (modularity decreased from > 0.7 to < 0.4), signaling that the development of bibliometric indicators had somewhat consolidated. This interpretation is supported when considering that, at about the same time, the 2^nd^ edition of the *Handbook of Quantitative Studies and Science and Technology Research* [60] and the influential textbook *Citation Analysis in Research Evaluation* [114] were published. Both books were published by Henk Moed, one of the leading scientists working at that time at one of the most central expert organizations in bibliometrics, the CWTS in Leiden, the Netherlands.

Fig 7 illustrates that Hirsch [7] turned the modularity curve of the JIF citation network around and up. Within a few years, from 2010 to 2015, it climbed to the same level from which it had started to decrease in the 1990s. In other words, the JIF citation network became internally fragmented and moved away from the path of gradual consolidation. As mentioned above, if JIF-related indicator development had continued to grow after 2005 at the same speed as before, only 8 new indicators would have been published instead of 47. Consequently, the 39 “additional” indicators, most of which (n = 25, Table 2) were contributed by peripheral and isolated organizations, increased the fragmentation of the JIF citation network considerably and almost in parallel with the HI citation network.

Thus, our empirical results suggest that limited reputational control is a characteristic structural feature of the research area of ECA. Our longitudinal inter-organizational network data show that it is not a coincidence that one of the two breakthroughs in the research area came from an outsider [7] because most of the new bibliometric indicators that have been published since the early 1970s came from organizations operating from peripheral and isolated positions in the research area. Moreover, the share of influential follow-up inventions from these quarters of the research area are as high as the research area’s central organizations’ share, and the percentage of newcomers is still higher than the percentage of those who have a track record.

## 5. Summary and discussion

This paper presents a method to empirically investigate the extent of reputational control in intellectual fields. This method consists of defining a set of comparable inventions within a circumscribed research area (i.e., novel contributions that are cognitively related) and then determining the origin of these inventions within a scientific collaboration network. The set of related inventions was defined as all citation impact metrics introduced since 1972. Based on the ECA review literature, we found two subsets, which we called “follow-up inventions” to the JIF and HI, with different dynamics in publication growth. The size of the scientific collaboration network was determined by all publications that cite any of the relevant follow-up inventions. Based on Whitley’s theory, we distinguished between inventions originating from the core, semi-core, periphery, and outsiders of this collaboration network as a way to characterize fields that are controlled by a core group of dedicated specialists (strong reputational control) versus fields that are not characterized by highly field-specific knowledge and competencies (low reputational control). Notably, our measure of reputational control does not constitute a measure of cognitive consensus. Competing claims could be raised in intellectual debates among specialists while their debate is inaccessible to methodological outsiders. In contrast, our empirical data indicate that ECA as a research area has developed little reputational control thus far, as many intellectual contributions, including the most influential ones, came from the periphery, and even from outsiders. We conclude from these findings that, though ECA can be described bibliometrically as a separate research area within the broader category of library and information sciences, the growth in the volume of publications has not been accompanied by the formation of an intellectual field with strong reputational control. This finding on the social structure of ECA is relevant for understanding the present state of professionalization in bibliometric evaluation techniques.

We think that our method could be generalized to other inventions and intellectual fields. Whitley [28] presented convincing theoretical arguments on the organization of work control in science and proposed a comparative framework for different disciplines. Yet, he never explained how reputational control could be operationalized and measured. Therefore, we make a case for more empirical studies on the theory of reputational control. Along the same lines, we argue that our measures of reputational control are complementary to other approaches studying social closure and cohesion in academic fields. For example, Moody [93] studied collaboration networks in sociology between 1975 and 1999 and found a highly cohesive co-authorship core that has increased in size over time. These findings fit well with Burris’ [115] study on PhD-exchange networks among U.S. sociology departments, showing that graduates from the top-20 departments account for 69% of the sociological faculty hired in all 94 PhD-granting departments. These results suggest that a stable set of prestigious university departments defines legitimate ways of doing sociology, enabling broad collaboration among scholars working in different subject areas. However, neither Moody [93] nor Burris [115] studied intellectual contributions in sociology. Therefore, it would be interesting to examine how their results square with those from the approach applied in this article. More specifically, it would be interesting to compare various measures of reputational control. Through the lens of Whitley’s theory, Burris’ faculty placements could be interpreted as a measure of reputational control and, thus, would be a suitable candidate for a comparison with our measures derived from inter-organizational citation networks.

We also offer here a new theoretical perspective of the ongoing debate on research metrics. Using Abbott’s theory of professions as a theoretical framework, we connect the observation of increasing demand for quantitative research assessment to the observation of competing social claims regarding how this increased demand for quantitative research assessment should be addressed without impairing basic functions of the science system. Therefore, through this theoretical lens, quantitative research assessments can be characterized as an arena for professional action. According to Abbott, competition among professional groups is profoundly shaped by national institutional settings. Therefore, we expect considerable variations regarding the professionalization of bibliometric expertise across different national science systems. Thus far, exclusive jurisdictions of bibliometric expertise have not been established, not even in countries with regular evaluation regimes for public research. Rather, bibliometric expertise appears to assume a status subsidiary to peer review [39]. Support for the adoption of bibliometric assessments on the levels of organizations and national systems is built around two arguments: substantially lower costs compared to comprehensive peer review exercises [44, 116] and increased transparency regarding evaluation criteria and honesty of peer review [114].

As mentioned above, a detailed historical reconstruction of the professional field of quantitative research evaluation has been provided for only the Netherlands [39]. Therefore, more detailed historical studies focusing on other national research systems are clearly needed. One important issue could be the historical analysis of countries in which national expert organizations are set up similar to the Netherlands, such as Norway’s Nordic Institute for Studies in Innovation, Research, and Education (NIFU), and where bibliometric indicators have been used in national research evaluations [117, 118]. These analyses could then be contrasted with countries where no bibliometric expert organizations emerged, such as the UK, and where the use of research metrics has remained contested [41, 44]. Once such historical studies are available, a more comprehensive triangulation of political-historical and scientific developments is possible, as well as a more fully developed sociological answer to the question examined here. In that sense, the results of this study are preliminary. A more comprehensive triangulation between historical case studies of particular countries with the empirical evidence presented here can be regarded as a fruitful avenue for further research.

Furthermore, we argue for a connection between Abbott’s theory of professions and Whitley’s theory of reputational control in order to clarify the relationship between academic work and professional work. The concept of reputational organizations analytically specifies how scientific work is different from and lends credibility to professional work that is characterized by Abbott as the application of abstract knowledge to complex individual cases. Professional experts are likely to convince actors in public and legal arenas to confer to them exclusive rights to provide their services in societally relevant problem areas when they draw upon knowledge from academic fields characterized by high reputational control (not consensus), including the control of highly specific knowledge, technical skills, and the standardization of their communication. The reason for this is that such academic fields provide scientific legitimacy and, thus, a valuable currency in the competition for professional jurisdictions. If our argument is valid, it would also hold the other way around: the role of specialized experts is contested, meaning that they have difficulties obtaining professional accreditation in public and legal arenas if they draw on knowledge from academic fields characterized by low reputational control because such knowledge confers little legitimacy and is a liability in the competition for professional jurisdictions.

Several prominent contributions make it clear that the application of bibliometrics to research evaluation is contested among scientists [3, 4, 43]. The present study goes beyond the simple statement of contestation, arguing that an organized scientific community that could lay claims to authoritative recommendations for professional evaluation practice has not yet emerged in the case of ECA. If reputational control is high, we would expect novel recommendations for improved research assessment to be launched by the field as a result of the recent intensification of research on this topic. Yet, in the present situation of limited reputational control, even carefully drafted academic contributions to improve citation analysis (i.e., follow-up inventions) appear unlikely to have a significant impact on research assessment practice.

Although the argument presented in this article provides empirical data in support of the conceptual framework mentioned above, we regard our findings basically as a first step to better understand the connection between structural features of academic fields and the professionalization of expertise. Within Abbott’s framework, there are alternative routes of how bibliometric assessment practices can become institutionalized despite the amorphous character of its academic sector. Instead of a hypothetical, well-defined research community disseminating universal professional standards through recognized institutional channels, case studies suggest that expert organizations play an important role in defining what currently counts as state of the art in bibliometric research assessment. These expert organizations offer contract research and consultancy that meets the aforementioned demands for performance assessment and accountability by research managers, administrators, and science policy. The Center for Science and Technology Studies (CWTS) at the University of Leiden is a case in point. Founded in the 1980s as a university research group pioneering bibliometric assessment methods in the context of national higher education and research policies, CWTS currently has clients from among public research organizations in several European countries [39]. The bibliometric methodology of CWTS serves as a model of legitimate practice that is frequently adopted by other bibliometric experts in Europe. However, despite considerable investment in an improved in-house database, these practices ultimately remain dependent on the citation data and reference standards that are available in large databases, such as the WoS or Scopus.

It should be clear from our analysis that we do not regard a strong professionalization of bibliometric expertise, in Abbott’s term a “full jurisdiction”, as a necessary or desirable development. Rather, we aim to improve our sociological understanding of how socio-cognitive field structures can either facilitate or curtail opportunities for bibliometric experts and expert organizations to achieve professional accreditation in national legal and public arenas. Problematic from a normative point of view is the overriding role of database providers in defining reference standards of research performance. Higher education rankings have been criticized on the grounds that non-experts gained control over the definition of what constitutes excellent education [119]. Clearly, the same argument applies to the definition of high-impact research as provided by Clarivate Analytics (WoS) and Elsevier (Scopus). As long as ECA in the academic sector remains a loosely defined group with little capacity for collective action, commercial database providers define de facto standards in research assessment. If our argument is valid, we would expect that bibliometric techniques will continue to be contested among scientific elites and face considerable hurdles to become firmly institutionalized in national science policies. Yet, this lack of support among scientific elites apparently does not prevent the spread of “desktop” bibliometrics offered by large commercial database providers and publishers. Clearly, in many public research organizations and funding bodies, such ready-made citation impact metrics provide relatively cheap solutions for routine performance assessment. Based on our results, we assume that these assessment practices will continue to spread and gain in influence as long as reputational control in ECA remains weak.

## Supporting information

S1 Table JIF-related follow-up inventions. S1 Table includes 74 JIF-related follow-up inventions and the original JIF publication (Garfield 1972). Compilation based on handbooks and review literature (Section 3.2). Citation frequency in Web of Science core collection from publication year until 12/31/2016, including articles, reviews, letters, notes, and proceedings.
(DOCX)

S2 Table HI-related follow-up inventions. S2 Table includes 95 HI-related follow-up inventions and the original HI publication (Hirsch 2005). Compilation based on handbooks and review literature (Section 3.2). Citation frequency in Web of Science core collection from publication year until 12/31/2016, including articles, reviews, letters, notes, and proceedings.
(DOCX)

S1 Movie Network evolution in JIF-related follow-up research, 1972–2016. The file shows the development of the inter-organizational citation network in JIF follow-up research based on moving 5-year citation windows. Data source: Web of Science.
(GIF)

S2 Movie Network evolution in HI-related follow-up research, 2005–2016. The file shows the development of the inter-organizational citation network in HI follow-up research based on moving 5-year citation windows. Data source: Web of Science.
(GIF)

S3 Movie Network evolution in total (JIF and HI) follow-up research, 1972–2016. The file shows the development of the inter-organizational citation network in total (JIF and HI) follow-up research based on moving 5-year citation windows. Data source: Web of Science.
(GIF)
