# Supplementary material for: Does bibliometric research confer legitimacy to research assessment practice? A sociological study of reputational control, 1972-2016
Source: PLoS One. 2018 Jun 14;13(6):e0199031. doi: 10.1371/journal.pone.0199031 (PMC6002049; doi:10.1371/journal.pone.0199031)
Supplement: S1 Table — includes 74 JIF-related follow-up inventions and the original JIF publication (Garfield 1972). Compilation based on handbooks and review literature (Section 3.2). Citation frequency in Web of Science core collection from publication year until 12/31/2016, including articles, reviews, letters, notes, and proceedings. (DOCX) [file pone.0199031.s001.docx]

**S1 Table. JIF-related follow-up inventions.**

| **ID: JIF-** | **Index name** | **Authors** | **PY** | **Title** | **Journal** | **Volume: pp.** | **Citation frequency** |
| --- | --- | --- | --- | --- | --- | --- | --- |
| 1 | Journal impact factor | Garfield E | 1972 | Citation analysis as a tool in journal evaluation | Science | 178: 471-479 | 1034 |
| 2 | Quality index for economic journals | Bush WC, Hamelman PW, Staaf RJ | 1974 | A quality index for economic journals | The Review of Economics and Statistics | 56: 123-125 | 43 |
| 3 | Influence weight | Pinski G, Narin F | 1976 | Citation influence for journal aggregates of scientific publicatinos: Theory, with application to the literature of physics | Information Processing & Management | 12: 297-312 | 262 |
| 4 | Discipline impact factor | Hirst G | 1978 | Discipline impact factors – method for determining core journal lists | J Ass Inf Sci Technol | 29: 171-172 | 82 |
| 5 | Corrected quality ratio | Lindsey D | 1978 | The corrected quality ratio: A composite index of scientific contribution to knowledge | Social Studies of Science | 8: 349-354 | 13 |
| 6 | Adjusted impact factor | Asai I | 1981 | Adjusted age distribution and its application to impact factor and immediacy index | J Ass Inf Sci Technol | 32: 172-174 | 22 |
| 7 | Consumption factor | Yanovsky VI | 1981 | Citation analysis significance of scientific journals | Scientometrics | 3: 223-233 | 27 |
| 8 | Standardized citation scores | McAllister PR, Narin F, Corrigan JG, | 1983 | Programmatic evaluation and comparison based on standardized citation scores | IEEE Transactions on engineering management | 30: 205-211 | 19 |
| 9 | Impact adjusted citations | Liebowitz S, Palmer J | 1984 | Assessing the relative impacts of economics journals | Journal of Economic Literature | 22: 77-88 | 228 |
| 10 | Relative indicators | Schubert A, Braun T | 1986 | Relative indicators and relational charts for comparative assessment of publication output and citation impact | Scientometrics | 9: 281-291 | 219 |
| 11 | Average subfield impact factor | Vinkler P | 1986 | Evaluation of some methods for the relative assessment of scientific publications | Scientometrics | 10: 157-177 | 86 |
| 12 | Characteristic scores and scales | Schubert A, Glänzel W, Braun T | 1987 | Subject field characteristic citation scores and scales for assessing research performance | Scientometrics | 12: 267-291 | 42 |
| 13 | Mean impact factors for subject categories | Vinkler P | 1988 | Bibliometric features of some scientific subfields and the scientometric consequences therefrom | Scientometrics | 14: 453-474 | 24 |
| 14 | Ellwein index, Faculty member score | Ellwein LB, Khachab M, Waldman RH | 1989 | Assessing research productivity: evaluating journal publication acrss academic departments | Academic medicine | 64: 319-325 | 19 |
| 15 | Normalized mean citation rate | Braun T, Glänzel W | 1990 | United Germany, the new scientific superpower? | Scientometrics | 19: 513-521 | 28 |
| 16 | Highly cited papers | Plomp P | 1990 | The significance of the number of highly cited papers as an indicator of scientific prolificacy | Scientometrics | 19: 185-197 | 30 |
| 17 | Standard journal impact | Vinkler P | 1991 | Possible causes of differences in information impact of journals from different subfields | Scientometrics | 20: 145-161 | 20 |
| 18 | Normalized impact factor | Sen BK | 1992 | Documentation note. Normalised impact factor | Journal of documentation | 48: 318-325 | 32 |
| 19 | Mean expected citation rate based on bibliographic coupling | Schubert A, Braun T | 1993 | Reference standards for citation based assessments | Scientometrics | 26: 21-35 | 33 |
| 20 | Mean field citation score mFCS | Moed HF, De Bruin RE & Van Leeuwen TN | 1995 | New bibliometric tools for the assessment of national research performance – database description, overview of indicators and first applications | Scientometrics | 33: 381-422 | 240 |
| 21 | Global journal impact | Egghe L, Rousseau R | 1996 | Average and global impact of a set of journals | Scientometrics | 36: 97-107 | 29 |
| 22 | Standard impact factor for fields of science | Marshakova-Shaikevich I | 1996 | The standard impact factor as an evaluation tool of science fields and scientific journals | Scientometrics | 35: 283-290 | 19 |
| 23 | Article vs. journal impact | Harter SP, Nisonger TE | 1997 | ISI's impact factor as misnomer: A proposed new measure to assess journal impact | J Ass Inf Sci Technol | 48: 1146-1148 | 27 |
| 24 | Journal diffusion factors | Rowlands I | 2002 | Journal diffusion factors: a new approach to measuring research influence | ASLIB Proceedings | 54: 77-84 | 31 |
| 25 | Top 1% and top 10% most highly cited papers | Tijssen RJW, Visser MS, Van Leeuwen TN | 2002 | Benchmarking international scientific excellence: Are highly cited papers an appropriate frame of reference? | Scientometrics | 54: 381-397 | 77 |
| 26 | Journal diffusion factors | Frandson TF | 2004 | Journal diffusion factors: a measure of diffusion? | ASLIB Proceedings | 56: 5-11 | 16 |
| 27 | Rank-normalized impact factor | Pudovkin, Garfield E | 2004 | Rank-normalized impact factor: A way to compare journal performance across subject categories | Proceedings of fhe 67th ASIS&TAnnual Meeting | 41: 507-515 | 18 |
| 28 | CHAL-impact factor | Sombatsompop T, Markpin W, Premkamolnetr N | 2004 | A modified method for calculating the Impact Factors of journals in ISI Journal Citation Reports: Polymer Science Category in 1997-2001 | Scientometrics | 60: 217-235 | 37 |
| 29 | General case of synchronous and diachronous impact factors | Frandsen TF, Rousseau R | 2005 | Article impact calculated over arbitrary periods | J Ass Inf Sci Technol | 56: 58-62 | 29 |
| 30 | Ratio to mathematics | Podlubny I | 2005 | Comparison of scientific impact expressed by the number of citations in different fields of science | Scientometrics | 64: 95-99 | 30 |
| 31 | Median and percentile impact factors | Rousseau R | 2005 | Median and percentile impact factors: A set of new indicators | Scientometrics | 63: 431-441 | 30 |
| 32 | SCEAS Rank | Sidiropoulos A, Manolopoulos Y | 2005 | A citation-based system to assist prize awarding | SIGMOD Rec | 34:54-60 | 15 |
| 33 | Impact factor point average (rank-normalized) | Sombatsompop T, Markpin W | 2005 | Making an equality of ISI impact factors for different subject fields | J Ass Inf Sci Technol | 56: 676-683 | 34 |
| 34 | Weighted PageRank | Bollen J, Rodriguez MA, Van de Sompel H | 2006 | Journal status | Scientometrics | 69: 669-687 | 154 |
| 35 | Balanced citation count | Sidiropoulos A, Manolopoulos Y | 2006 | Generalized comparison of graph-based ranking algorithms for publications and authors | Journal of Systems and Software | 79: 1679-1700 | 20 |
| 36 | Relative superiority coefficient | Hu XJ | 2007 | Relative superiority coefficient of papers: a new dimension for institutional research performance in different fields | Scientometrics | 72: 389-402 | 0 |
| 37 | Citation z-score | Lundberg J | 2007 | Lifting the crown – citation z-score | Journal of informetrics | 1: 145-154 | 111 |
| 38 | CiteRank | Walker D, Xie H, Yan KK, Maslov S | 2007 | Ranking scientific publications using a model of network traffic | Journal of statistical mechanics | 06: P06010 | 24 |
| 39 | Journal Eigenfactor and article influence score | Bergstrom CT, West JD, Wieseman MA | 2008 | The Eigenfactor metrics | The journal of neuroscience | 28: 11433-11434 | 82 |
| 40 | SJR scimago journal rank | Falagas ME, Kouranos VD, Arencibia-Jorge R, Karageogopoulos DE | 2008 | Comparison of SCImago journal rank indicator with journal impact factor | The FASEB journal | 22: 2623-2628 | 101 |
| 41 | Weighed impact factor | Habibzadeh Yadollahie | 2008 | Journal weighted impact factor: a proposal | Journal of informetrics | 2: 164-172 | 18 |
| 42 | PageRank value | Ma N, Guan J, Zhao Y | 2008 | Bringing PageRank to the citation analysis | Information processing & management | 44: 800-810 | 67 |
| 43 | Paper quality index | Qiu J, Ma R, Cheng N | 2008 | New exploratory work of evaluating a researcher´s output | Scientometrics | 77: 335-344 | 2 |
| 44 | Audience factor | Zitt M, Small H | 2008 | Modifying the journal impact factor by fractional citation weighting: The audience factor | J Ass Inf Sci Technol | 59: 1856-1860 | 96 |
| 45 | J factor | Ball R, Mittermaier B, Tunger D | 2009 | Creation of journal-based publication profiles of scientific institutions – a methodology for the interdisciplinary comparison of scientific research based on the J-factor | Scientometrics | 81: 381-392 | 9 |
| 46 | Science author rank algorithm SARA | Radicchi F, Fortunato S, Markines B, Vespignani A | 2009 | Diffusion of scientific credits and the ranking of scientists | Physical Review E | 80: 056103 | 106 |
| 47 | Current contribution index | Vinkler P | 2009 | Introducing the Current Contribution Index for characterizing the recent, relevant impact of journals | Scientometrics | 79: 409-420 | 7 |
| 48 | Fractional citation count index | Leydesdorff L, Opthof T | 2010 | Scopus’s source normalized impact per paper (SNIP) versus a journal impact factor based on fractional counting of citations | J Ass Inf Sci Technol | 61: 2365-2369 | 51 |
| 49 | Source normalized impact per paper SNIP | Moed HF | 2010 | Measuring contextual citation impact of scientific journals | Journal of informetrics | 4: 265-277 | 157 |
| 50 | Robust variant of audience factor | Zitt M | 2010 | Citing-side normalization of journal impact: A robust variant of the Audience Factor | Journal of informetrics | 4: 392-406 | 35 |
| 51 | Scientific strength | Abramo G, Cicero T, D´Angelo CA | 2011 | A field-standardized application of DEA to national-scale research assessment of universities | Journal of informetrics | 5: 618-628 | 17 |
| 52 | Low- and high impact indicators | Albarran P, Ortuno I, Ruiz-Castillo J | 2011 | The measurement of low- and high-impact in citation distributions: technical results. | Journal of informetrics | 5: 48-63 | 20 |
| 53 | Aggregated citations of cited articles ACCA | Bharathi DG | 2011 | Methodology for the evaluation of scientific journals: Aggregated citations of cited articles | Scientometrics | 86: 563-574 | 5 |
| 54 | Axiomatic analysis of impact factors | Bouyssou D, Marchant T | 2011 | Bibliometetric rankings of journals based on Impact Factors: An axiomatic approach | Journal of informetrics | 5: 75-86 | 18 |
| 55 | f value | Fragkiadaki E, Evangelidis G, Samaras N, Dervos DA | 2011 | f-Value: measuring an article´s scientific impact | Scientometrics | 86: 671-686 | 3 |
| 56 | Successful papers | Kosmulski M | 2011 | Successful papers: a new idea in evaluation of scientific output | Journal of informetrics | 5: 481-485 | 14 |
| 57 | Integrated impact indicators (I3) | Leydesdorff L, Bornmann L | 2011 | Integrated impact indicators compared with impact factors: an alternative research design with policy implications | J Ass Inf Sci Technol | 62: 2133-2146 | 61 |
| 58 | R(i,k) | Leydesdorff L, Bornmann, L, Mutz R, Opthof T | 2011 | Turning the tables on citation analysis one more time: principles for comparing sets of documents | J Ass Inf Sci Technol | 62: 1370-1381 | 81 |
| 59 | Theoretical influence function | Ravallion M, Wagstaff A | 2011 | On measuring scholarly influence by citations | Scientometrics | 44: 1-22 | 22 |
| 60 | PrestigeRank | Su C, Pan YT, Zhen YN et al. | 2011 | PrestigeRank: A new evaluation method for papers and journals | Journal of informetrics | 5: 1-13 | 6 |
| 61 | Citation distribution score CDS index | Vinkler P | 2011 | Application of the distribution of citations among scientific publications in scientometric evaluations | J Ass Inf Sci Technol | 62: 1963-1978 | 19 |
| 62 | Mean normalized citation score MNCS | Waltman L, van Eck NJ, van Leeuwen TN, Visser MS, van Raan AFJ | 2011 | Towards a new crown indicator: some theoretical considerations | Journal of informetrics | 5: 37-47 | 115 |
| 63 | Recursive field-normalized bibliometric performance indicator | Waltman L, Yan E, van Eck NJ | 2011 | A recursive field-normalized bibliometric performance indicator: an application to the field of library and information science | Scientometrics | 89: 301-314 | 20 |
| 64 | P-Rank in heterogeneous networks | Yan E, Ding Y, Sugimoto CR | 2011 | P-Rank: An indicator measuring prestige in  heterogeneous scholarly networks | J Ass Inf Sci Technol | 61: 609-614 | 24 |
| 65 | Article impact index (AII) | Abramo G, Cicero T, D’Angelo CA | 2012 | Revisiting the scaling of citations for research assessment | Journal of informetrics | 6: 470-479 | 28 |
| 66 | Success index | Franceschini F, Galetto M, Maisano D, Mastrogiacomo L | 2012 | The success-index: an alternative approach to the h-index for evaluating an individual's research output | Scientometrics | 92: 621-641 | 16 |
| 67 | SJR2 scimago journal rank | Guerrero-Bote VP, Moya-Anegón F | 2012 | A further step forward in measuring journals' scientific prestige: The SJR2 indicator | Journal of informetrics | 6: 674-688 | 39 |
| 68 | Total influence index | Liu JS, Lu LYY, Ho MHC | 2012 | Total influence and mainstream measures for scientific researchers | Journal of informetrcs | 6: 496-504 | 3 |
| 69 | Journal integrated impact index | Ma T, Wang GF, Dong K, Cao M | 2012 | The Journal´s Integrated Impact Index: a new indicator for journal evaluation | Scientometrics | 9: 649-658 | 0 |
| 70 | Two-parameter reverse engineering | Radicchi F, Castellano C | 2012 | A reverse engineering approach to the suppression of citation biases reveals universal properties of citation distributions | PLOS one | 7: e33833 | 35 |
| 71 | Average weights of tied papers | Schreiber M | 2012 | Inconsistencies of recently proposed citation impact indicators and how to avoid them | J Ass Inf Sci Technol | 63: 2062-2073 | 8 |
| 72 | Exchange rates | Crespo JA, Li Y, Ruiz-Castillo J | 2013 | The Measurement of the Effect on Citation Inequality of Differences in Citation Practices across Scientific Fields | PLOS one | 8: e58727 | 13 |
| 73 | Credibility formula | Pérez-Hornero P, Aria-Nicolás JP, Pulgarin AA, Pulgarin A | 2013 | An annual JCR impact factor calculation based on Bayesian credibility formulas | Journal of informetrics | 7: 1-9 | 1 |
| 74 | Assigning publications fractionally to percentile intervals | Waltman L, Schreiber M | 2013 | On the calculation of percentile based bibliometric indicators | J Ass Inf Sci Technol | 64: 372-379 | 33 |
| 75 | Revised SNIP | Waltman L, van Eck NJ, van Leeuwen TN, Visser MS | 2013 | Some modifications to the SNIP journal impact indicator | Journal of informetrics | 7: 272-285 | 34 |

S1 Table includes 74 JIF-related follow-up inventions and the original JIF publication (Garfield 1972). Compilation based on handbooks and review literature (Section 3.2). Citation frequency in Web of Science core collection from publication year until 12/31/2016, including articles, reviews, letters, notes, and proceedings.
