# Supplementary material for: Does bibliometric research confer legitimacy to research assessment practice? A sociological study of reputational control, 1972-2016
Source: PLoS One. 2018 Jun 14;13(6):e0199031. doi: 10.1371/journal.pone.0199031 (PMC6002049; doi:10.1371/journal.pone.0199031)
Supplement: S2 Table — includes 95 HI-related follow-up inventions and the original HI publication (Hirsch 2005). Compilation based on handbooks and review literature (Section 3.2). Citation frequency in Web of Science core collection from publication year until 12/31/2016, including articles, reviews, letters, notes, and proceedings. (DOCX) [file pone.0199031.s002.docx]

**S2 Table. HI-related follow-up inventions.**

| **ID: HI-** | **Index name** | **Authors** | **PY** | **Title** | **Journal** | **Volume: pp.** | **Citation frequency** |
| --- | --- | --- | --- | --- | --- | --- | --- |
| 1 | Hirsch Index | Hirsch JE | 2005 | An index to quantify an individual's scientific research output | Proceedings of the national academy of sciences of the USA PNAS | 102(46): 16569-16572 | 1978 |
| 2 | Taber c-index | Taber DF | 2005 | Quantifying publication impact | Science | 309: 2166 | 12 |
| 3 | Hb index | Banks MG | 2006 | An extension of the Hirsch index: Indexing scientific topics and compounds | Scientometrics | 69:161-168 | 70 |
| 4 | hI index | Batista P, Campiteli M, Kinouchi O, et al. | 2006 | Is it possible to compare researchers with different scientific interests? | Scientometrics | 68:179-189 | 197 |
| 5 | Hirsch-type index for journals | Braun T, Glänzel W, Schubert A | 2006 | A hirsch-type index for journals | Scientometrics | 69: 169–173 | 206 |
| 6 | g-index | Egghe L | 2006 | Theory and practice of the g-index | Scientometrics | 69: 131–152 | 498 |
| 7 | h index sequence and h index matrix | Liang L | 2006 | h-Index sequence and h-index matrix: Constructions and applications | Scientometrics | 69: 153-159 | 54 |
| 8 | Prathap h2 index | Prathap G | 2006 | Hirsch-type indices for ranking institutions’ scientific research output | Current Science | 91: 1439 | 51 |
| 9 | Residual h | Symonds MRE, Gemmell NJ, Braisher TL, Gorringe KL, Elgar MA | 2006 | Gender differences in publication output: Towards an unbiased metric of research performance | PLOS one | 1: e127 | 50 |
| 10 | h rate | Burrell QL | 2007 | Hirsch index or Hirsch rate? Some thoughts arising from Liang’s data | Scientometrics | 73:19-28 | 50 |
| 11 | Dynamic h-index | Egghe L | 2007 | Dynamic h-index: The Hirsch index in function of time | J Ass Inf Sci Technol | 58: 452-454 | 41 |
| 12 | Rescaling h index | Iglesias JE, Pecharroman C | 2007 | Scaling the h-index for different scientific ISI fields | Scientometrics | 73: 303-320 | 81 |
| 13 | R index and AR index | Jin BH, Liang LM, Rousseau R, Egghe L | 2007 | The R and AR-indices: complementing the h-index | Chinese Science Bulletin | 52: 855-863 | 202 |
| 14 | Successive h indices | Schubert A | 2007 | Successive h-indices | Scientometrics | 70: 201-205 | 59 |
| 15 | Contemporary h-index, trend h-index, normalized h-index | Sidiropoulos A, Katsaros D, Manopoulos Y | 2007 | Generalized hirsch index for disclosing latent facts in citation networks | Scientometrics | 72: 253–280 | 115 |
| 16 | Tapered h-index (h(T)) | Anderson T, Hankin R, Killworth P | 2008 | Beyound the durfee square. Enhancing the h-index to score total publication output | Scientometrics | 76: 577–588 | 50 |
| 17 | IQp index | Antonakis J, Lalive R | 2008 | Quantifying Scholarly Impact: IQp Versus the Hirsch h | J Ass Inf Sci Technol | 59:956–969 | 28 |
| 18 | m index | Bornmann L, Mutz R, Daniel H | 2008 | Are there better indices for evaluation purposes than the h-index? A comparison of nine different variants of the h-index using data from biomedicine | J Ass Inf Sci Technol | 59: 830–837 | 158 |
| 19 | hw index | Egghe L, Rousseau R | 2008 | An h-index weighted by citation impact | Information processing & management | 44: 770-780 | 34 |
| 20 | Concatenated h index | Glänzel W | 2008 | H-index concatenation | Scientometrics | 77: 369-72 | 7 |
| 21 | Discounted cumulative impact index, DCI | Järvelin K, Persson O | 2008 | The DCI-index: Discounted cumulated impact based research evaluation | J Ass Inf Sci Technol | 59: 1433-1440 | 9 |
| 22 | hm impact index | Molinari JF, Molinari A | 2008 | A new methodology for ranking scientific institutions | Scientometrics | 75: 163–174 | 52 |
| 23 | Generalization of h index based on indicator cf | Radicchi F, Fortunato S, Castellano C | 2008 | Universality of citation distributions: toward an objective measure of scientific impact | PNAS | 105: 17268–17272 | 230 |
| 24 | v index | Riikonen P, Vihinen M | 2008 | National research contributions: a case study on Finnish biomedical research | Scientometrics | 77: 207–222 | 8 |
| 25 | AR^2^ index | Rousseau R, Jin BH | 2008 | The age dependent h-type AR^2^ index: Basic properties and a case study | J Ass Inf Sci Technol | 59: 2305-11 | 6 |
| 26 | h-type index | Rousseau R, Rons N | 2008 | Another h-type index for institutional evaluation | Current Science | 95: 1103 | 1 |
| 27 | Dynamic h type index | Rousseau R, Ye FY | 2008 | A proposal for a dynamic h-type index | J Ass Inf Sci Technol | 59: 1853-55 | 22 |
| 28 | Successive rational h indices | Ruane F, Tol RSJ | 2008 | Rational (successive) h-indices: An application to economics in the Republic of Ireland. | Scientometrics | 75: 395–405 | 47 |
| 29 | hm index | Schreiber M | 2008 | A modification of the h-index: The h(m)-index accounts for multy-authored manuscripts | Journal of Informetrics | 2: 211-216 | 57 |
| 30 | Rational successive g index | Tol RSJ | 2008 | A rational, successive g-index applied to economics departments in Ireland | Journal of Informetrics | 2: 149-155 | 26 |
| 31 | hα index, Gα index | Van Eck NJ, Waltman L | 2008 | Generalizing the h- and g-indices | Journal of Informetrics | 4: 263–271 | 50 |
| 32 | b index | Brown RJC | 2009 | A simple method for excluding self-citation from the h-index: the b-index | Online Information Review | 33: 1129-1136 | 5 |
| 33 | Generalized Kosmulski indices | Deineko VG, Woeginger GJ | 2009 | A new family of scientific impact measures: The generalized Kosmulski-indices | Scientometrics | 80: 819-826 | 11 |
| 34 | Generalization of h index, w index, maxprod indices | Gagolewski M, Grzegorzewski P | 2009 | A geometric approach to the construction of scientific impact indices | Scientometrics | 81: 617-634 | 8 |
| 35 | multidim h2, multidim h3 | Garcia-Perez MA | 2009 | A multidimensional extension to Hirsch's h-index | Scientometrics | 81: 779-785 | 21 |
| 36 | h rat, g rat/ h_r_ real -valued h and g indices | Guns R, Rousseau R | 2009 | Real and rational variants of the h-index and the g-index | Journal of Informetrics | 3: 64–71 | 30 |
| 37 | f index coterminal citations : unrelated to h index | Katsaros D, Akritidis L, Bozanis P | 2009 | The f Index: Quantifying the Impact of Coterminal Citations on Scientists’ Ianking | J Ass Inf Sci Technol | 60: 1051-56 | 11 |
| 38 | H per decade hpd | Kosmulski M | 2009 | New seniority independent h-type index | Journal of Informetrics | 3: 341-347 | 9 |
| 39 | History h-index | Randic | 2009 | Citations versus limitations of citations: beyond Hirsch index | Scientometrics | 80: 809-818 | 10 |
| 40 | hms indicator | Schreiber M | 2009 | The influence of self-citation corrections and the fractionalised counting of multi-authored manuscripts on the Hirsch index | Annalen der Physik | 18: 607-621 | 17 |
| 41 | Single publication h-index | Schubert A | 2009 | Using the h-index for assessing single publications | Scientometrics | 78: 559-565 | 24 |
| 42 | Modified impact index MII | Sypsa V, Hatzakis A | 2009 | Assessing the impact of biomedical research in academic institutions of disparate sizes | BMC Medical research methodology | 9: 33 | 21 |
| 43 | f index, t index | Tol RSJ | 2009 | The h-index and its alternatives. An application to the 100 most prolific economists | Scientometrics | 80: 317-324 | 30 |
| 44 | π index | Vinkler P | 2009 | The p-index: a new indicator for assessing scientific impact | Journal of Information Science | 35:602-612 | 35 |
| 45 | Generalized g index | Woeginger GJ | 2009 | Generalizations of a's g index | J Ass Inf Sci Technol | 60: 1267-1273 | 11 |
| 46 | w index, wohlin index | Wohlin C | 2009 | A new index for the citation curve of researchers | Scientometrics | 81: 521-533 | 9 |
| 47 | e index | Zhang CT | 2009 | The e-index. Complementing the h-index for excess citations | PLOS one | 4: e5429 | 102 |
| 48 | ch index | Ajiferuke I, Wolfram D | 2010 | Citer analysis as a measure of research impact: Library and information science as a case study. | Scientometrics | 83:623–638 | 13 |
| 49 | hg index | Alonso S, Cabrerizo F, Herrera-Vidma E, Herrera F | 2010 | hg-index: A new index to characterize the scientific output of researchers based on the h- and g-indices | Scientometrics | 82:391-400 | 46 |
| 50 | Citation speed index | Bornmann L, Daniel HD | 2010 | The citation speed index: A useful bibliometric indicator to add to the h index | Journal of Informetrics | 4: 444-446 | 7 |
| 51 | h2 lower, h2 center, h2 upper | Bornmann L, Mutz R, Daniel HD | 2010 | The h index research output measurement: Two approaches to enhance its accuracy | Journal of Informetrics | 4: 404-417 | 22 |
| 52 | q^2^-index | Cabrerizo F J, Alonso S, Herrera-Viedma E, et al. | 2010 | q^2^-index: Quantitative and qualitative evaluation based on the number and impact of papers in the h-core | Journal of Informetrics | 4:23-28 | 28 |
| 53 | s index | de Visscher A | 2010 | An index to measure a scientist's specific impact | J Ass Inf Sci Technol | 61: 319-328 | 5 |
| 54 | CSS based h and g indices | Egghe L | 2010 | Characteristic scores and scales based on h-type indices | Journal of Informetrics | 4: 14-22 | 8 |
| 55 | Characterization of ch-index | Franceschini F, Maisano D, Perotti A, Proto A | 2010 | Analysis of the ch-index: an indicator to evaluate the diffusion of scientific research output by citers | Scientometrics | 85: 203-217 | 12 |
| 56 | Generalized h-index, generalized g-index | Glänzel W, Schubert A | 2010 | Hirsch-type characteristics of the tail of distributions. The generalised h-index | Journal of Informetrics | 4: 118-123 | 10 |
| 57 | hbar | Hirsch JE | 2010 | An index to quantify an individual’s scientific research output that takes into account the effect of multiple coauthorship | Scientometrics | 85: 741-754 | 58 |
| 58 | h-maj | Hu X, Rousseau R, Chen J | 2010 | In those fields where multiple authorship is the rule, the h-index should be supplemented by role-based h-indices | Journal of information science | 36: 73-85 | 21 |
| 59 | hfg-index | Kosmulski M | 2010 | Hirsch-type approach to the 2nd generation citations | Journal of Informetrics | 4: 257-264 | 4 |
| 60 | n index | Namazi MR, Fallahzadeh MK | 2010 | n-index: A novel and easily-calculable parameter for comparison of researchers working in different scientific fields | Indian journal of dermatology, venerology and leprology | 76: 229-230 | 3 |
| 61 | Mock h index | Prathap G | 2010 | Is there a place for a mock h-index? | Scientometrics | 84: 153-165 | 31 |
| 62 | Pi(v)-index | Vinkler P | 2010 | The pi(v)-index: a new indicator to characterize the impact of journals | Scientometrics | 82: 461-475 | 21 |
| 63 | wu index | Wu Q | 2010 | The w-index a measure to assess scientific impact by focussing on widely cited papers | J Ass Inf Sci Technol | 61:609-614 | 34 |
| 64 | tail-core ratio, v index, k index | Ye FY, Rousseau R | 2010 | Probing the h-core: An investigation of the tail-core ratio for rank distributions | Scientometrics | 84: 431-439 | 19 |
| 65 | Weighted he and hp indizes | Abbas AM | 2011 | Weighted indices for evaluating the quality of research with multiple authorship | Scientometrics | 88:107–131 | 10 |
| 66 | q index | Bartneck C, Kokkelmans S | 2011 | Detecting h-index manipulation through self-citation analysis | Scientometris | 87:85–98 | 50 |
| 67 | c index | Bras-Amoros M, Domingo-Ferrer J, Torra V | 2011 | A bibliometric index based on the collaboration distance between cited and citing authors | Journal of Informetrics | 5: 248–264 | 11 |
| 68 | Carbon h-index | Carbon CC | 2011 | The Carbon_h-Factor: Predicting Individuals' Research Impact at Early Stages of Their Career | PLOS one | 6: e28770 | 3 |
| 69 | Central area index & central interval index | Dorta-Gonzalez P, Dorta-Gonzalez MI | 2011 | Central indexes to the citation distribution: a complement to the h-index | Scientometrics | 88: 729-745 | 12 |
| 70 | Indirect h-index | Egghe L | 2011 | The single publication H-index and the indirect H-index of a researcher | Scientometrics | 88: 1003-1004 | 4 |
| 71 | First citation speed index fcsi | Egghe L, Bornmann L, Guns R | 2011 | A proposal for a first-citation-speed-index | Journal of Informetrics | 5: 181-186 | 4 |
| 72 | Regularity indices | Franceschini F, Maisano D | 2011 | Proposals for evaluating the regularity of a scientist’s research output | Scientometrics | 88: 279-295 | 5 |
| 73 | Fractional gh index | Galam S | 2011 | Tailor based allocations for multiple authorship: a fractional gh-index | Scientometrics | 89:365–379 | 12 |
| 74 | GS, GM, HS, HM indicators based on h index | Hu X, Rousseau R, Chen J | 2011 | On the definition of forward and backward citation generations | Journal of Informetrics | 5: 27-36 | 14 |
| 75 | Shape descriptors (c-descriptor and t- descriptor) | Kuan CH, Huang MH, Chen DZ | 2011a | Ranking patent assignee performance by h-index and shape descriptors | Journal of Informetrics | 5: 303-312 | 9 |
| 76 | Centroids of h-core and h-tail | Kuan CH, Huang MH, Chen DZ | 2011b | Positioning research and innovation performance using shape centroids of h-core and h-tail | Journal of Informetrics | 5: 515-528 | 9 |
| 77 | Harmonic p-indices | Prathap G | 2011 | The fractional and harmonic p-indices for multiple authorship | Scientometrics | 86: 239-244 | 2 |
| 78 | j index | Todeschini R | 2011 | The j-index: a new bibliometric index and multivariate comparisons between other common indices | Scientometrics | 87: 621–639 | 12 |
| 79 | h(nf)-indicator | Vieira ES, Gomes JANF | 2011 | An impact indicator for researchers | Scientometrics | 89: 607-629 | 7 |
| 80 | h index divided by the number of decades | Abt HA | 2012 | A publication index that is independent of age | Scientometrics | 91: 105-115 | 8 |
| 81 | Cumulative performance indices | Kozak M, Bornmann L | 2012 | A New Family of Cumulative Indexes for Measuring Scientific Performance | PLOS one | 7: e47679 | 3 |
| 82 | Total influence index, mainstream index | Liu JS, Lu LYY, Ho MSH | 2012 | Total influence and mainstream measures for scientific researchers | Journal of Informetrics | 6: 496-504 | 3 |
| 83 | Modified h index and modified g index | Liu XZ, Fang H | 2012 | Fairly sharing the credit of multi-authored papers and its application in the modification of h-index and g-index | Scientometrics | 91: 37–49 | 18 |
| 84 | Stochastic h index | Nair GM, Turlach BA | 2012 | The stochastic h-index | Journal of Informetrics | 6: 80-87 | 4 |
| 85 | Radius R of circular citation area | Sangwal K | 2012a | On the relationship between citations of publication output and Hirsch index h of authors: conceptualization of tapered Hirsch index hT, circular citation area radius R and citation acceleration a | Scientometrics | 93:987-1004 | 8 |
| 86 | α index | Sangwal K | 2012b | On the age-independent publication index | Scientometrics | 91:1053–1058 | 3 |
| 87 | Partnership ability index ϕ | Schubert A | 2012 | A Hirsch-type index of co-author partnership ability | Scientometrics | 91:303–308 | 20 |
| 88 | k-index, w-index | Anania G, Caruso A | 2013 | Two simple new bibliometric indexes to better evaluate research in disciplines where publications typically receive less citations | Scientometrics | 96: 617-631 | 1 |
| 89 | co-author core | Ausloos M | 2013 | A scientometrics law about co-authors and their ranking: the co-author core | Scientometrics | 95:895–909 | 18 |
| 90 | Profit p index | Aziz NA, Rozing MP | 2013 | Profit (p)-Index: The Degree to Which Authors Profit from Co-Authors | PLOS one | 8: e59814 | 10 |
| 91 | AB index, PR index | Biswal AK | 2013 | An Absolute Index (Ab-index) to Measure a Researcher’s  Useful Contributions and Productivity | PLOS one | 8: e84334 | 5 |
| 92 | CAV indicator | Egghe L, Guns R, Rousseau R | 2013 | Measuring co-authors’ contribution to an article’s visibility | Scientometrics | 95:55–67 | 7 |
| 93 | Year based h type indices | Mahbuba D, Rousseau R | 2013 | Year-based h-type indicators | Scientometrics | 96: 785-797 | 4 |
| 94 | Field-independent index, fi index | Wu J | 2013 | Investigating the universal distributions of normalized indicators and developing field-independent index | Journal of informetrics | 7: 63-71 | 2 |
| 95 | hIa index | Harzing AW, Alakangas S, Adams D | 2014 | hIa: an individual annual h-index to accommodate disciplinary and career length differences | Scientometrics | 99:811–821 | 14 |
| 96 | WL index | Wan X, Liu F | 2014 | WL-Index: Leveraging Citation Mention Number to Quantify an Individual’s Scientific Impact | J Ass Inf Sci Technol | 65: 2509–2517 | 2 |

S2 Table includes 95 HI-related follow-up inventions and the original HI publication (Hirsch 2005). Compilation based on handbooks and review literature (Section 3.2). Citation frequency in Web of Science core collection from publication year until 12/31/2016, including articles, reviews, letters, notes, and proceedings.
