# Supplementary figures and images for: Does bibliometric research confer legitimacy to research assessment practice? A sociological study of reputational control, 1972-2016
Source: PLoS One. 2018 Jun 14;13(6):e0199031. doi: 10.1371/journal.pone.0199031 (PMC6002049; doi:10.1371/journal.pone.0199031)

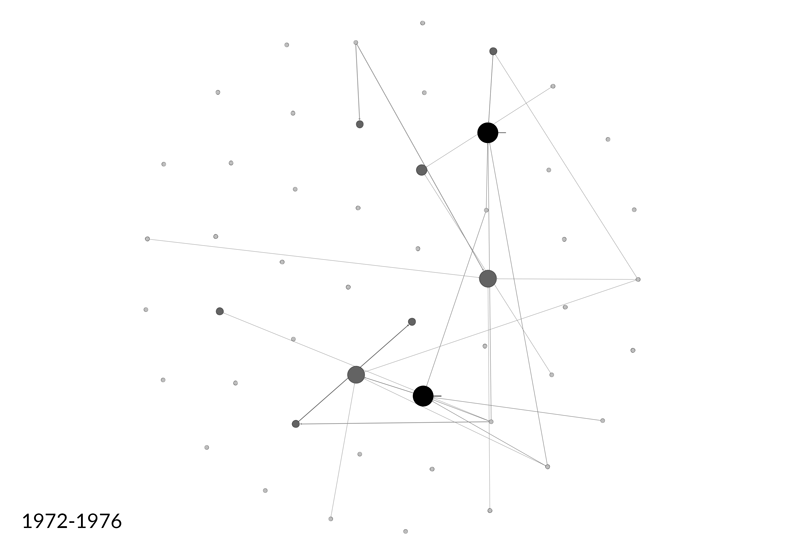

Supplement: S1 Movie — The file shows the development of the inter-organizational citation network in JIF follow-up research based on moving 5-year citation windows. Data source: Web of Science. (GIF) [file pone.0199031.s003.gif]

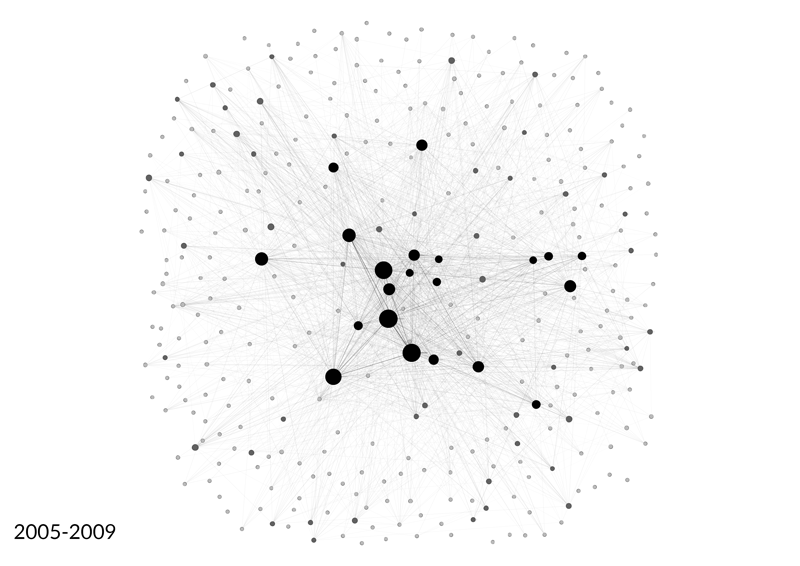

Supplement: S2 Movie — The file shows the development of the inter-organizational citation network in HI follow-up research based on moving 5-year citation windows. Data source: Web of Science. (GIF) [file pone.0199031.s004.gif]

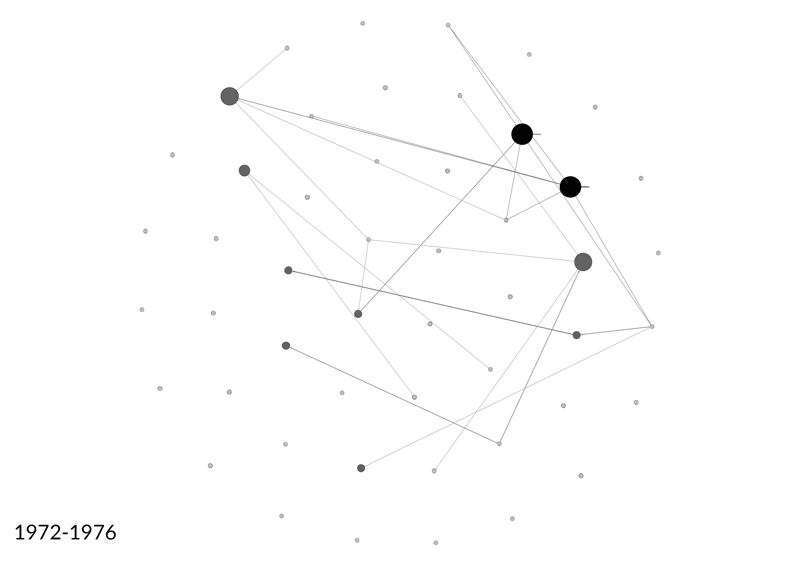

Supplement: S3 Movie — The file shows the development of the inter-organizational citation network in total (JIF and HI) follow-up research based on moving 5-year citation windows. Data source: Web of Science. (GIF) [file pone.0199031.s005.gif]
